# Cyclic Ion Mobility for Hydrogen/Deuterium Exchange-Mass Spectrometry Applications

**DOI:** 10.1021/acs.analchem.3c05753

**Published:** 2024-04-01

**Authors:** Damon Griffiths, Malcolm Anderson, Keith Richardson, Satomi Inaba-Inoue, William J. Allen, Ian Collinson, Konstantinos Beis, Michael Morris, Kevin Giles, Argyris Politis

**Affiliations:** † Faculty of Biology, Medicine and Health, School of Biological Sciences, The University of Manchester, Manchester M13 9PT, United Kingdom; ‡ Manchester Institute of Biotechnology, University of Manchester, Princess Street, Manchester M1 7DN, United Kingdom; § Waters Corporation, Stamford Avenue, Altrincham Road, Wilmslow SK9 4AX, United Kingdom; ∥ Department of Life Sciences, Imperial College London, Exhibition Road, South Kensington, London SW7 2AZ, United Kingdom; ⊥ Rutherford Appleton Laboratory, Research Complex at Harwell, Oxfordshire, Didcot OX11 0FA, United Kingdom; # Diffraction and Scattering Division, Japan Synchrotron Radiation Research Institute, SPring-8, 1-1-1, Kouto, Sayo, Hyogo 679-5198, Japan; ∇ School of Biochemistry, University of Bristol, Bristol BS8 1TD, United Kingdom

## Abstract

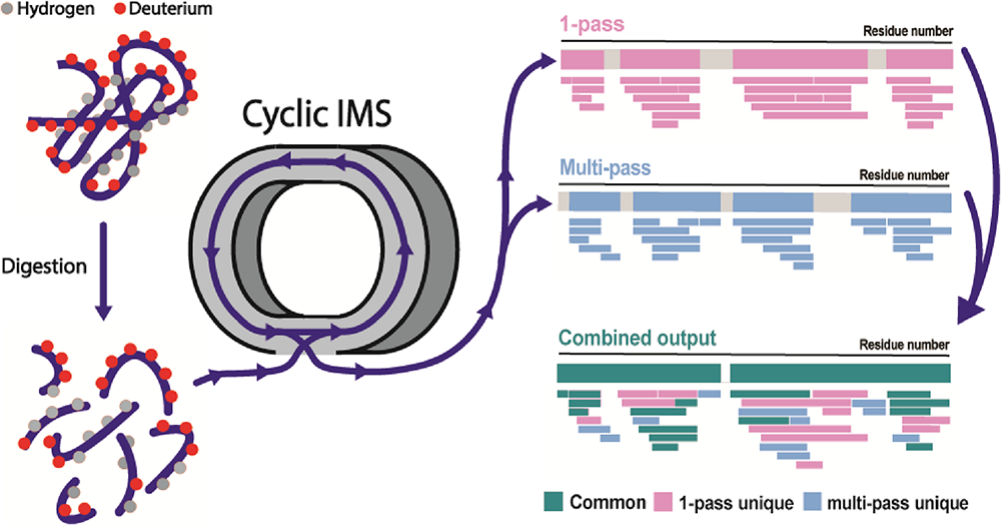

Hydrogen/deuterium exchange-mass spectrometry (HDX-MS) has emerged as a powerful tool to probe protein dynamics. As a bottom-up technique, HDX-MS provides information at peptide-level resolution, allowing structural localization of dynamic changes. Consequently, the HDX-MS data quality is largely determined by the number of peptides that are identified and monitored after deuteration. Integration of ion mobility (IM) into HDX-MS workflows has been shown to increase the data quality by providing an orthogonal mode of peptide ion separation in the gas phase. This is of critical importance for challenging targets such as integral membrane proteins (IMPs), which often suffer from low sequence coverage or redundancy in HDX-MS analyses. The increasing complexity of samples being investigated by HDX-MS, such as membrane mimetic reconstituted and *in vivo* IMPs, has generated need for instrumentation with greater resolving power. Recently, Giles et al. developed cyclic ion mobility (cIM), an IM device with racetrack geometry that enables scalable, multipass IM separations. Using one-pass and multipass cIM routines, we use the recently commercialized SELECT SERIES Cyclic IM spectrometer for HDX-MS analyses of four detergent solubilized IMP samples and report its enhanced performance. Furthermore, we develop a novel processing strategy capable of better handling multipass cIM data. Interestingly, use of one-pass and multipass cIM routines produced unique peptide populations, with their combined peptide output being 31 to 222% higher than previous generation SYNAPT G2-Si instrumentation. Thus, we propose a novel HDX-MS workflow with integrated cIM that has the potential to enable the analysis of more complex systems with greater accuracy and speed.

## Introduction

Hydrogen/deuterium exchange-mass spectrometry (HDX-MS) is a well-established methodology for probing protein higher-order structure in solution.^1^ To achieve this, HDX-MS measures the time-dependent exchange of amide hydrogens of the polypeptide backbone with deuterium in the surrounding solvent, a reaction termed HDX. The HDX rate in folded proteins is primarily dependent on hydrogen bonding status.^2^ Consequently, changes in HDX-MS readout can report on a variety of protein behavioral characteristics such as conformational dynamics,^3^ folding pathways,^4^ protein–protein and protein–small molecule interactions,^5^ and epitope mapping for biopharmaceutical characterization.^6^ In a typical HDX-MS experiment, proteins are first incubated in deuterated buffer to induce spontaneous deuterium incorporation into the backbone, followed by reaction quenching by dropping solution pH and temperature to 2.5 and 0–1 °C, respectively. Online proteolytic digestion is then performed, followed by separation of peptides at low temperature via reverse-phase liquid chromatography (LC) and detection via mass spectrometry (MS). This “bottom-up” LC-MS approach allows changes in HDX-MS readout to be localized to specific structural regions of the protein at peptide-level resolution.^1,7^ Therefore, the quality of bottom-up HDX-MS data is largely determined by the number of peptides that are identified and monitored throughout the LC-MS experiment.

One caveat of classical HDX-MS workflows is the need to keep LC separations short and under quench conditions to minimize deuterium/hydrogen back-exchange.^8^ This, in turn, limits available LC-MS peak capacity and can often result in insufficient detection and identification of peptides, thereby providing low sequence coverage and redundancy of the target protein(s). This is particularly problematic for challenging targets such as G protein-coupled receptors (GPCRs) and other integral membrane proteins (IMPs), which often require lengthy screening and optimization of HDX-MS conditions to obtain high-quality results.^9,10^ Several different approaches have been employed to improve peptide identification.^10−15^ One example is ion mobility (IM),^16^ a technique that separates gas-phase ions based on their mobilities through an inert buffer gas under the influence of an electric field.^17,18^ IM can operate on the millisecond time scale. Thus, its use in bottom-up LC-MS can provide an orthogonal dimension of peptide separation without increasing the length of the experiment.

The widespread use of IM has been primarily driven by the commercialization of the SYNAPT line of quadrupole time-of-flight (QTOF) mass spectrometers by Waters Corporation. These instruments contain an integrated traveling wave ion mobility (TWIM) cell with linear geometry, which permits contiguous IM separations throughout bottom-up LC-MS experiments.^19^ For peptide identification and sequence mapping, SYNAPT instruments can perform MS^E^ data acquisition, whereby the instrument collision energy (CE) is periodically switched between low-energy and high-energy states to provide alternate scans of intact precursor and corresponding fragment (product) ions.^20^ Thus, peptide precursors are identified via a retention time (RT)-assisted alignment with their associated products. When TWIM is activated, high-definition MS^E^ (HDMS^E^) can be performed, whereby MS^E^ is performed but with contiguous IM-based separation of precursor ions prior to fragmentation. Thus, for HDMS^E^, drift time (DT) alignment can be performed in addition to RT alignment, thereby providing a higher-accuracy precursor-product matching. Consequently, HDMS^E^ has previously been shown to improve data quality in HDX-MS and other bottom-up “omics” applications.^21,22^ HDMS^E^ also allows ions with the same RT to be separated based on DT. Thus, processed spectra can be generated with reduced complexity based on their DT extraction, which can, in turn, facilitate the identification and monitoring of the isotopic distribution for a peptide of interest.^21,23^ This is particularly beneficial for HDX-MS analyses where short chromatographic gradients often result in coeluting peptides with overlapping spectra. Despite these improvements, the increasing complexity of samples being investigated by HDX-MS, such as mimetic reconstituted and *in vivo* IMPs, has generated need for instrumentation with greater resolving power.^24^

Several approaches have been employed to increase IM's resolving power.^25−30^ One creative approach to increasing drift tube length without drastically increasing the instrument footprint was cyclotron IM developed by Clemmer and co-workers.^31−33^ The device consisted of four curved drift tube segments arranged in a cyclic geometry through which ions could be separated over multiple passes. Thus, the overall effective drift tube length could be “dialed up” by increasing the number of device passes. This work was later supplemented by Giles et al. in 2019 with the development of cyclic ion mobility (cIM), a TWIM separator with a “racetrack” geometry capable of scalable, multipass separations.^34^ Using the reverse-sequence pentapeptides SDGRG and GRGDS, they demonstrated that the cIM resolving power increases, as expected, with the square root of the number of device passes. Furthermore, unlike Clemmer’s cyclotron, cIM allows numerous ions of different mobilities to simultaneously undertake multiple device passes before ejection, making it ideally suited for the analysis of complex proteolytic digests. Thus, via extended IM separations, cIM has potential to offer improved LC-MS peak capacity in bottom-up LC-MS applications such as HDX-MS. The design, operation, and multifunctional capabilities of cIM have been described in detail.^34,35^ A handful of publications have now used the SELECT SERIES Cyclic IMS with cIM technology for the purpose of increasing LC-MS peak capacity during peptide mapping and other omics applications.^36−39^ Despite this, to date, no systematic evaluation of cIM in HDX-MS has been performed nor has its multipass functionality been utilized online in any bottom-up LC-MS or omics application; only one-pass routines have previously been reported.

Here, using both one-pass and, for the first time, multipass cIM, we evaluated Cyclic IMS performance when analyzing challenging IMP targets in the HDX-MS context. Using four model IMP samples (i.e., frizzled class GPCR smoothened receptor (SMO), secondary active transporter XylE, ATP binding cassette transporter MsbA, and the heterotrimeric SecYEG complex), we benchmarked Cyclic IMS performance relative to previous generation SYNAPT G2-Si instrumentation and report its enhanced performance. Manual inspection of multipass data revealed high-quality peptide ions not captured by default HDMS^E^ processing methods, which we demonstrate is likely a consequence of cyclic wrap-around. To address this, we developed a novel cIM data processing approach capable of better handling multipass data. Interestingly, the use of one-pass and multipass cIM routines resulted in unique peptide populations, with their combined output being up to 222 and 37% higher than SYNAPT G2-Si instrumentation and Cyclic IMS with one-pass cIM, respectively. Thus, we propose a novel HDX-MS workflow that utilizes combined one-pass/multipass peptide mapping to further increase LC-MS peak capacity beyond use of one pass alone. We envision that adoption of this approach in HDX-MS and other bottom-up LC-MS and omics applications has potential to improve analyses of complex samples in the future.

## Experimental Section

### Materials

Facade-EM detergent was purchased from Avanti Polar Lipids (Alabaster, Alabama) and *n*-dodecyl-β-d-maltopyranoside (DDM) was purchased from Anatrace (Maumee, Ohio). Unless otherwise stated, all other chemicals and reagents were purchased from Sigma-Aldrich (Gillingham, Dorset). Sample expression and purification methods are outlined in the Supporting Information.

### Peptide Mapping and Hydrogen/Deuterium Exchange (HDX) Sample Handling

HDX sample handling and mixing were performed using an automated Trajan HDX PAL system (LEAP Technologies, Carrboro). For nondeuterated peptide mapping, 5 μL of 20 μM XylE, 8.32 μM SMO, 21.4 μM MsbA, or 4 μM SecYEG was diluted in equilibration buffer (XylE: 27.5 μL of 10 mM potassium phosphate + 0.02% DDM at pH 7.4; SMO: 27.5 μL of 50 mM HEPES + 200 mM NaCl + 0.03%/0.003% DDM/CHS at pH 7.4; MsbA: 95 μL of 20 mM Tris + 150 mM NaCl + 0.02% facade-EM at pH 7.4; SecYEG: 95 μL of 20 mM Tris + 50 mM KCl + 0.02% DDM at pH 8). For deuterium exchange, samples were diluted in deuterated equivalents of their respective equilibration buffers in identical volumes for 1 min. After exchange, samples were diluted 1:1 in quench buffer (XylE: 100 mM potassium phosphate + 0.1% DDM at pH 2.3; SMO: 100 mM potassium phosphate + 100 mM TCEP + 4 M urea + 0.1% DDM at pH 2.3; MsbA: 100 mM potassium phosphate + 4 M urea at pH 2.3; SecYEG: 0.7% formic acid + 0.1% DDM at pH 2.3) at 1 °C for 15 s. Quenched samples were then injected (60 μL of XylE and SMO, 100 μL of MsbA and SecYEG) into a 50 μL sample loop and passed through a BEH Enzymate column (Waters Corporation, Wilmslow) containing immobilized porcine pepsin at 20 °C. For nondeuterated control samples, four replicates were performed while HDX measurements were performed in triplicate.

### Liquid Chromatography–Mass Spectrometry with Cyclic Ion Mobility

All LC-cIM-MS experiments were performed on a SELECT SERIES Cyclic IM QTOF instrument coupled to an M-class nanoACQUITY UPLC system and an HDX manager (Waters Corporation, Wilmslow). After digestion, peptides underwent trapping/desalting using a BEH C_18_ VanGuard precolumn (Waters Corporation, Wilmslow) at 100 μL/min for 3 min in mobile phase A (0.2% formic acid in HPLC grade H_2_O), followed by an 8 min UPLC separation at 40 μL/min using a 1 mm × 100 mm BEH C_18_ analytical column (Waters Corporation, Wilmslow) with an 8–55% gradient of mobile phase B (0.2% formic acid in HPLC grade acetonitrile). All trapping and chromatography were performed at 1 °C to minimize deuterium/hydrogen back-exchange.

The electrospray ionization source was operated in positive mode, and QTOF was operated in resolution V-mode with HDMS^E^ enabled for data-independent acquisition. The MS was calibrated with sodium iodide, and leucine enkephalin was used as lock mass for postacquisition mass accuracy correction. Spectra were acquired between 50 and 2000 *m*/*z* with capillary voltage 3.0 kV, sample cone 20 V, and transfer CE ramping from 15 to 50 V. For one-pass cIM, a 10 ms injection, 3 ms separation, and 34 ms ejection/acquire sequence was used with a TW static height of 23 V and an analog-to-digital converter (ADC) start delay of 13 ms and using two pushes per bin. For multipass cIM, a 10 ms injection, 18.13 ms separation, and 34 ms ejection/acquire sequence was used with a TW static height of 22 V and an ADC start delay of 28 ms and using two pushes per bin. To minimize peptide carryover, the Enzymate column was washed with pepsin wash (1.5 M Gu-HCl, 0.4% MeOH, 0.5% formic acid, pH 3) and a sawtooth LC gradient was performed between sample injections.

### Liquid Chromatography–Mass Spectrometry with Linear Ion Mobility

For experiments using linear TWIM, the method outlined above was performed but with a SYNAPT G2-Si QTOF instrument (Waters Corporation, Wilmslow). Identical UPLC equipment, buffers, samples, and columns were used to maximize comparability. Further details regarding instrument tuning and mobility parameters can be found in the Supporting Information.

### Data Analysis and Visualization

For peptide mapping, nondeuterated HDMS^E^ files were processed via ProteinLynx Global Server (PLGS) ver. 3.0.2 (Waters Corporation, Wilmslow) with low energy and elevated energy count thresholds of 250 and 100, respectively. The primary digest reagent was set to nonspecific with 0 missed cleavages and no false discovery rate filter. Peak lists were database searched against the target protein(s) and pepsin sequences. For manual drift full width at half-maximum (FWHM) trendline input, the Apex3D/Peptide3D executables were operated via batch file in the windows command prompt (templates can be found in the Supporting Information). Identical parameters were used but with the addition of manually inputted drift FWHM-start and -end arguments. Peak lists were then database searched against target protein(s) and pepsin sequences by importing spectra to PLGS.

PLGS search results and deuterium exchange measurements were then imported into DynamX ver. 3.0 (Waters Corporation, Wilmslow). To minimize inclusion of false positive and/or nonquantifiable peptides, previously optimized peptide threshold values were applied.^40^ This included a minimum intensity of 1000, a minimum sequence length of 5, a maximum sequence length of 30, minimum products of 2, minimum average products per amino acid of 0.11, minimum consecutive products of 1, minimum sum intensity for products of 472, a minimum PLGS score of 6.62, and a minimum precursor MH+ error of 5 ppm. Furthermore, peptides were retained only if identified in three out of four replicates with an RT RSD of ≤4%. All peptides were manually validated on DynamX by using default settings. For combined one-pass/multipass data, one-pass and multipass DynamX output files were concatenated and redundancies removed to create “combined” cluster files. DynamX cluster data and heat maps were then exported to Deuteros ver. 2.0^41^ and the HDX-Viewer web server^42^ for data interpretation and visualization.

## Results and Discussion

### Benchmarking Cyclic IMS Performance Relative to SYNAPT G2-Si

**Figure 1:**
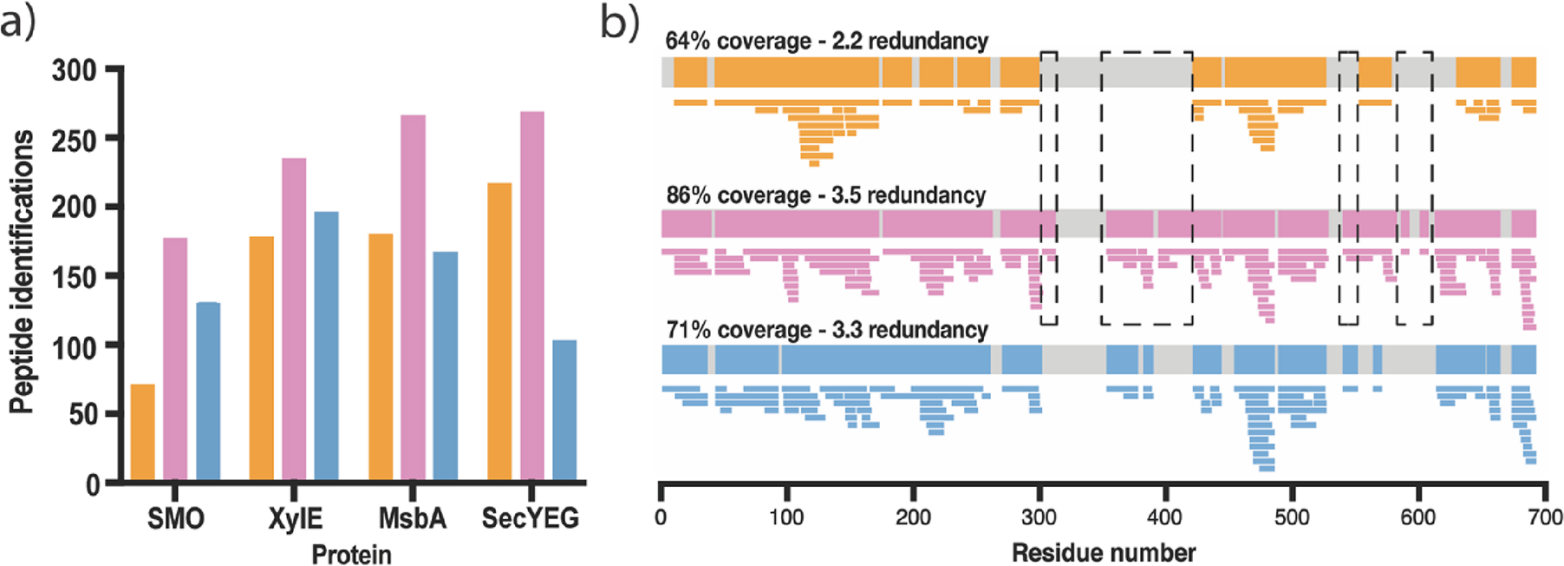
(a) Number of peptide identifications for SMO, XylE, MsbA, and SecYEG, using SYNAPT G2-Si (orange), Cyclic IMS one-pass (pink), and Cyclic IMS multipass (blue). (b) Peptide coverage maps for SMO using SYNAPT G2-Si (orange), cyclic one-pass (pink), and cyclic multipass (blue). Dashed boxes represent sequence segments not captured on SYNAPT G2-Si. Coverage and redundancy values were taken from Deuteros.^41^

To evaluate Cyclic IMS performance, nondeuterated peptide mapping was performed and benchmarked relative to previous generation SYNAPT G2-Si instrumentation with linear TWIM technology (Figure 1). To maximize comparability, identical batches of purified protein and buffers were used, as well as identical UPLC equipment including analytical, trapping, and digestion columns.

When using one-pass, cIM operates such that all ions pass through the device once, irrespective of their size and charge. However, owing to the device’s longer 98 cm path length, one-pass cIM still theoretically provides higher resolution than its 25 cm linear counterpart.^34,43^ As expected, when compared to the SYNAPT G2-Si, use of Cyclic IMS with one-pass cIM provided peptide identification increases of 144, 32, 48, and 24% for SMO, XylE, MsbA, and SecYEG, respectively (Figure 1a), which translated into increased sequence coverage and/or redundancy for all targets (Figure S1 and Table S1). For SMO, this led to a 22% increase in sequence coverage, thereby permitting capture of coverage in functionally important domains (ICL2, TMD4, ECL2, TMD6, and ECL3) that were previously omitted during HDX-MS optimization efforts on the SYNAPT G2-Si (Figure 1b). This demonstrates that use of Cyclic IMS can increase the information content of bottom-up LC-MS data when analyzing challenging IMP targets. For XylE, MsbA, and SecYEG, less pronounced increases in sequence coverages were observed (Table S1), likely a result of their coverages already being high on SYNAPT G2-Si. Nevertheless, the increased peptide identification afforded by one-pass cIM also led to 59, 10, 30, and 14% increases in redundancy for SMO, XylE, MsbA, and SecYEG, respectively, thereby increasing sequence resolution (Table S1). Thus, overall, Cyclic IMS with one-pass cIM routine provided substantial improvement in data quality under identical HDX-MS conditions.

In contrast to one-pass, multipass cIM operates such that all ions circumnavigate the device repeatedly until their ejection after a specified time window has elapsed. Therefore, owing to the variability in peptide size, shape, and charge, the number of device passes per peptide is highly variable and can be upscaled by increasing the duration of the separation. Here, an 18.13 ms cIM separation was applied, with number of passes for peptides likely ranging in the low to midsingle digit range.^44^ During peptide mapping, application of multipass cIM consistently underperformed relative to one-pass (Figure 1a). When compared to SYNAPT G2-Si, multipass cIM provided 84 and 10% increases in peptide identification for SMO and XylE, respectively, but decreases of 7 and 52% for MsbA and SecYEG. This was counterintuitive, as the extended mobility separation was expected to increase peptide identification owing to its increased resolving power.

To understand why Cyclic IMS with one-pass cIM outperformed the SYNAPT G2-Si, MsbA raw data were manually inspected to assess key instrument performance attributes (Figure S2). In the SYNAPT G2-Si data, ions reaching detector saturation were observed (Figure S2a,b), potentially limiting the accessible sample dynamic range and interfering with algorithmic peptide identification. Despite some ion intensities being 10-fold higher, reduced detector saturation was observed in one-pass cIM data (Figure S2a,b), likely owing to its upgraded ADC detector with improved dynamic range and linear response at high ion currents. While enabling dynamic range enhancement on SYNAPT G2-Si instrumentation can mitigate detector saturation, it doubles scan times.^21^ Thus, the use of Cyclic IMS appears to reduce saturation without compromising on chromatographic resolution. To compare sensitivity in the absence of saturation, monoisotopic peak intensities were manually measured for nine MsbA peptides and compared (Figure S2c). Here, all nine peptides from one-pass cIM exhibited intensity increases between 82 and 457% relative to their SYNAPT equivalents. This is consistent with previously published findings^36^ and is likely a significant factor contributing to the observed increase in peptide identification. The one-pass cIM data also showed a marked increase in precursor-product matching across all targets, thereby resulting in a greater proportion of peptides passing DynamX filtering (Figure S2d). Owing to the DT-aligned nature of precursor-product matching in HDMS^E^, this increase is likely a consequence of the instrument’s higher mobility resolution. Therefore, increased sensitivity, dynamic range, and mobility resolution all likely contribute to enhanced peptide identification. However, the updated collision cell design in the Cyclic IMS instrument may also contribute. Nevertheless, these results underscore the potential of Cyclic IMS with one-pass cIM to improve data quality under identical conditions.

To understand why multipass cIM underperformed relative to one-pass, we also compared the MsbA multipass cIM data. Interestingly, multipass showed less pronounced intensity increases (5–331%) than one-pass, with one peptide even being lower than its SYNAPT G2-Si equivalent (Figure S2c). This suggests that multipass ion intensity increases are more peptide-specific, potentially owing to the increased transmission path length for peptides that undergo high numbers of passes.^34^ Thus, it is possible that ion loss over multiple passes could contribute to the decreased peptide identification. Despite its extended mobility separation, multipass cIM also provided decreased precursor-product matching relative to one-pass (Figure S2d). Owing to the identical CE ramp performed when using one-pass and multipass cIM, differences in fragment generation are unlikely to account for this difference. To assess whether high-quality peptide spectra were present in multipass cIM data but omitted during PLGS processing, the SecE one-pass cIM peptide database was used to search for peptides in the SecE multipass cIM data (Figure S2e). Despite only two peptides being identified via PLGS, spectra for 26 peptides were observed across all SecE cIM multipass LC-MS replicates. Moreover, many of these peptides were of high intensity (i.e., >1e5), suggesting that decreased intensity is not the only factor reducing peptide identification in multipass experiments. When comparing the SecE one-pass and multipass PLGS outputs, peptide ions were consistently identified across the one-pass LC-MS replicate, but this reduced in the multipass despite being observed manually across replicate (Figure S2f). Therefore, dropping DynamX threshold parameters could not improve results without introducing large quantities of false positive identifications. These findings, coupled with the observed decrease in precursor-product matching, led to the hypothesis that PLGS software provided suboptimal identification and/or alignment of target ions in cIM multipass data, as opposed to instrumental issues at the LC-MS level. We aimed to address this by developing a novel processing strategy for improved handling of multipass cIM data.

### Differential Drift FWHM Trendline Processing for Multipass cIM Peptide Mapping

**Figure 2:**
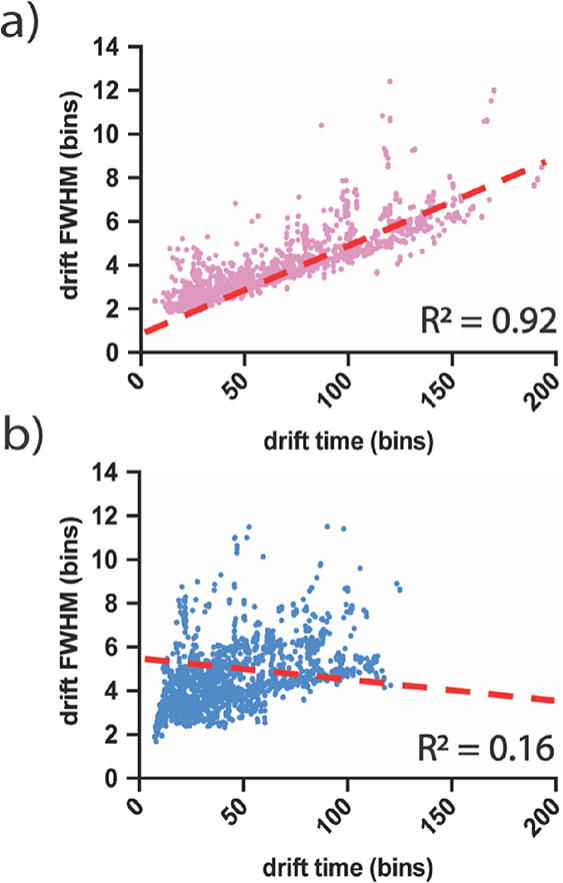
Measured DT bin number vs drift FWHM values for the top 2000 most intense ions detected in XylE one-pass data (a) and XylE multipass data (b). Dashed red lines indicate Apex3D-autocalculated drift FWHM trendlines with *R*^2^ value at the bottom right of the box.

Due to diffusion, larger ions with longer DTs typically have broader arrival time distributions compared to their higher-mobility counterparts in linear TWIM separations.^45^ This leads to a positive correlation between DT and drift peak FWHM when separating molecules of single isomeric species.^46^ Because it operates under the same principle, the one-pass cIM also exhibits this characteristic (Figure 2a and Figure S3). In contrast, multipass cIM has the potential to exhibit “wrap-around” phenomena, whereby slower ion populations are overtaken by speedier ions within the cyclic mobility device over multiple passes.^44^ Thus, depending on the relative geometric positioning of each ion population when ejection from the device is triggered, it is possible for slower ions with longer periodic DTs to exit the device ahead of speedier ions with shorter periodic DTs. Consequently, in multipass cIM data, the linear positive correlation between DT and drift FWHM is lost (Figure 2b and Figure S3), as ions with any drift FWHM can potentially be found in any DT bin. Moreover, it should be noted that in multipass experiments of this nature, ions subjected to more passes tend to occupy a narrower “DT” range, leading to an apparent bias toward earlier DTs. Despite this, DT vs *m*/*z* graphs (Figure S4) of the same data illustrate that multipass experiments still distribute ions more evenly across the two-dimensional DT-*m*/*z* space, thereby enhancing separation for ions undergoing multiple passes in DT.

Prior to peak detection, the PLGS Apex3D algorithm smooths IM data using a kernel smoother. For appropriate selection of smoothing kernel, approximate determination of drift FWHM is required, as smoothing wide peaks with narrow kernels results in undersmoothing and retention of noise (resulting in peak splitting) while smoothing narrow peaks with wide kernels results in oversmoothing (and merging of adjacent peaks). Rather than measuring all drift peak widths individually, Apex3D autonomously samples ∼700 extracted ion mobiligrams (EIMs) and, using a linear regression model, calculates a drift FWHM versus DT trendline to approximate peak width at any given DT bin. Owing to the linear relationship between DT and drift FWHM in one-pass data, a single compromise trendline with a high *R*^2^ value is sufficient to provide appropriate smoothing of all IM data (Figure 2a). However, for cIM multipass data, the wrap-around-derived loss of direct relationship between DT and drift FWHM results in trendlines with low positive correlation and *R*^2^ values (Figure 2b). Based on this observation, we hypothesized that Apex3D-autocalculated trendlines are insufficient to appropriately smooth all data across the multipass mobility distribution and that manual input of trendlines to accommodate wider drift FWHM at earlier DTs could improve results. For Apex3D processing, autocalculated trendlines can be overridden via manual input of “driftFWHM-start” and “driftFWHM-end” arguments, which define the drift FWHM trendline values at DT bin 1 and bin 200, respectively. For example, a “1–4” trendline refers to a trendline with a drift FWHM value of 1 at DT bin 1 and a drift FWHM value of 4 at DT bin 200.

**Figure 3:**
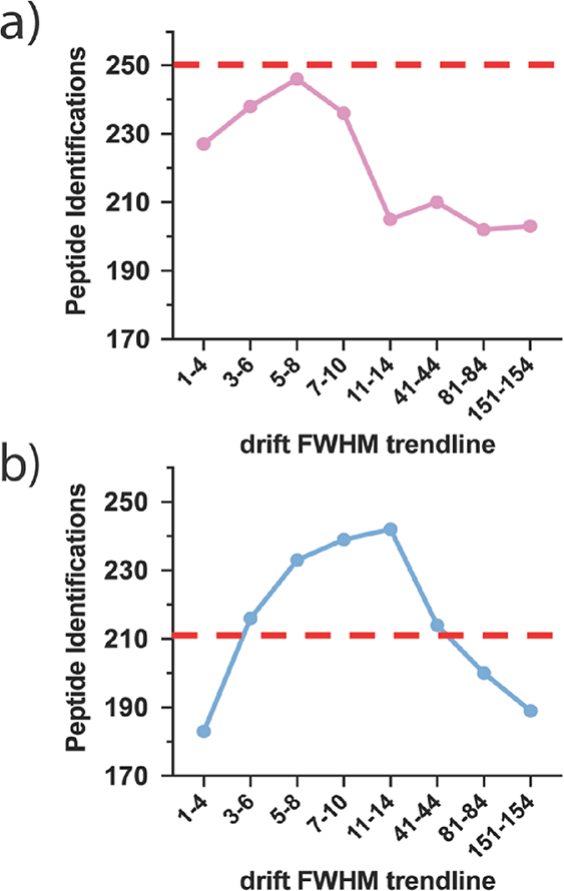
Number of peptide identifications for different drift FWHM trendlines applied on XylE one-pass data (a) and XylE multipass data (b) prior to manual curation. Dashed red lines indicate the number of peptide identifications obtained using a default autocalculated trendline.

To test this hypothesis, Apex3D processing was performed on XylE multipass and one-pass cIM data using eight variations of manually inputted trendlines and compared with default processing (Figure 3a,b). Initially, a 1–4 drift FWHM trendline was applied to the XylE multipass data to assess the effect of trendlines set below all detected peaks (Figure 3b). As expected, the 1–4 trendline resulted in a 13% reduction in identification relative to default processing: likely owing to increased undersmoothing of IM data. The trendline was then sequentially shifted upward throughout the mobility distribution to assess the effect of using increasingly wide kernel smoothers. Moreover, the trendline gradient was kept consistent and relatively flat to provide similar degrees of kernel smoothing throughout the mobility distribution within each processing variant. Raising the drift FWHM trendline to 3–6, 5–8, 7–10, and 11–14 resulted in peptide identification increases of 2, 10, 13, and 15%, respectively (Figure 3b). While the increase in 3–6 and 5–8 was expected (as they more closely resemble the autocalculated trendline), the further increases in 7–10 and 11–14 suggest that oversmoothing of multipass cIM data is less detrimental than undersmoothing. This is likely due to the high proportion of unimodal distributions that are seen in EIMs from less complex samples (e.g., purified proteins). Consequently, the detrimental effects from adjacent peak merging appear limited relative to what would likely be seen in more complex samples. Despite this, a further increase of trendline to 41–44, 81–84, and 151–154 resulted in sequential decrease (Figure 3b), thereby demonstrating that oversmoothing can still negatively impact peptide identification if trendlines are set too high. Thus, the 11–14 trendline (and similar trendlines within this region) appears optimal in accommodating wider drift FWHM at earlier DTs without detrimentally oversmoothing multipass IM data. As such, we advise employing trendlines that sit just above all detected ions in multipass mobility distributions to minimize undersmoothing while maintaining a minimal degree of oversmoothing for less complex data.

In contrast to multipass cIM, use of identical drift FWHM trendlines on XylE one-pass cIM data resulted in decreasing peptide identifications ranging from 2 to 19% relative to default processing (Figure 3a). This suggests that, due to the positive correlation between DT and drift FWHM in one-pass data, the autocalculated trendline is optimal and that manual modification only serves to detrimentally increase oversmoothing and/or undersmoothing of one-pass cIM data. This is further exemplified by the fact that the 3–6 and 5–8 trendlines, which most closely resemble the autocalculated trendline, were the variants with the highest numbers of peptide identification. Consequently, Apex3D-autocalculated trendlines were applied to all one-pass cIM data.

### Combining One-Pass and Multipass cIM Peptide Mapping Can Improve Sequence Coverage and Redundancy

**Figure 4:**
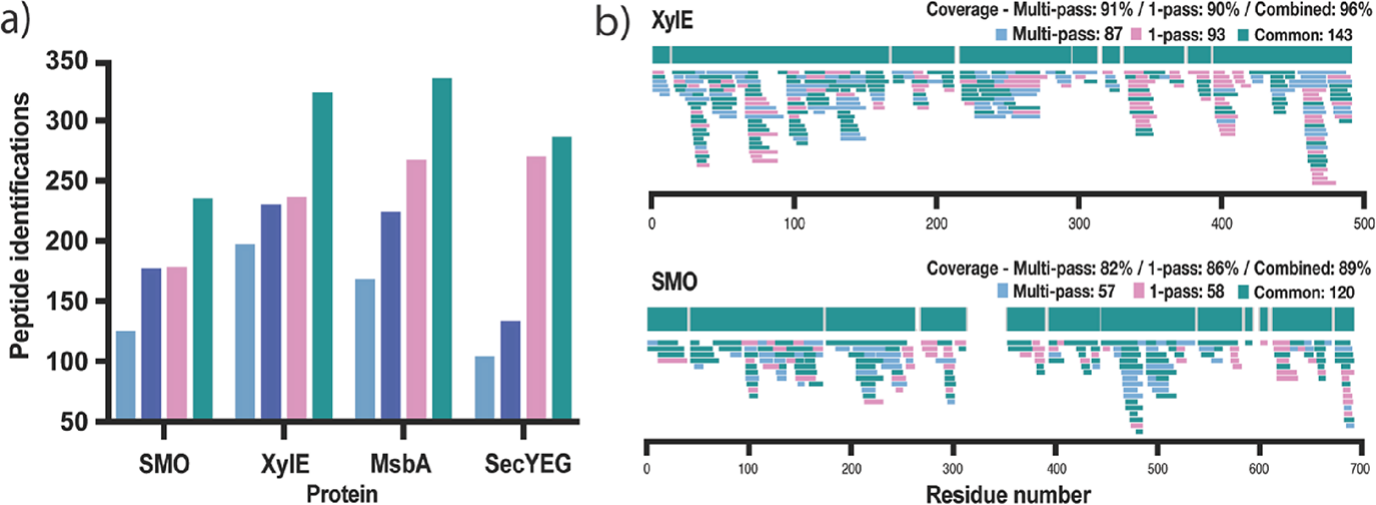
(a) Number of peptide identifications for SMO, XylE, MsbA, and SecYEG using cyclic multipass with autocalculated trendline (blue), cyclic multipass with the 11–14 trendline (dark blue), cyclic one-pass with autocalculated trendline (pink), and the combined one-pass/multipass peptide output (green). (b) Comparative peptide coverage map of XylE and SMO. Common peptides are in green, while peptides unique to multipass and one-pass are in blue and pink, respectively.

To further improve peptide mapping results, the 11–14 trendline was applied to the previously obtained multipass cIM peptide mapping data and compared to autocalculated trendlines (Figure 4a). Application of 11–14 trendlines increased the number of peptide identifications by 42, 17, 33, and 28% for SMO, XylE, MsbA, and SecYEG, respectively, thereby including a total of 170 peptides that would otherwise have been omitted using autocalculated trendlines. Thus, these results demonstrate the potential for alternative trendlines to improve multipass cIM peptide mapping results. While MsbA and SecYEG peptide identifications were still lower than their one-pass equivalents, SMO and XylE exhibited peptide identification numbers that were comparable. This increase also translated into increased coverage or redundancy for all assessed targets (Table S1).

Interestingly, use of one-pass and multipass cIM resulted in ion populations containing both common and unique peptides. Consequently, one-pass and multipass DynamX cluster data output were merged to generate comparative one-pass/multipass peptide maps (Figure 4b and Figure S5). When compared to data acquired on the SYNAPT G2-Si instrument, the combined one-pass/multipass peptide output exhibited increases of 222, 80, 85, and 31% for SMO, XylE, MsbA, and SecYEG, respectively (Figure 4a and Table S1), Thus, while one-pass cIM outperformed multipass as an individual cIM routine, use of one-pass and multipass in combination can further improve overall performance in bottom-up LC-MS applications beyond use of one-pass cIM alone (Figure S6). The increased peptide identification afforded by combined one-pass/multipass cIM analyses further increased sequence coverage and/or redundancy for all assessed targets (Figure S5 and Table S1), leading to 96 and 89% sequence coverages for XylE and SMO, respectively.

Owing to the identical CE ramp used during one-pass and multipass cIM peptide mapping, differences in fragment generation are unlikely to account for the unique peptide populations observed. Inspection of the raw one-pass and multipass PLGS databases revealed that, while peptide identification was highly reproducible across LC-MS replicates for common peptides, the unique peptide populations showed markedly decreased reproducibility under the opposing cIM routine (Figure S7a), with many falling below the set threshold. Thus, a lack of reproducible identification across replicate appears to be a significant factor contributing to the unique peptide populations observed. Moreover, reduced precursor–product matching was also observed for unique peptides under the opposing cIM routine (Figure S7b). While this alone does not directly push most peptides below the set threshold, it has a knock-on effect in reducing average products per amino acid and consecutively matched products (Figure S7c,d). Thus, less efficient precursor–product matching under the opposing cIM routine also appears to contribute. One possible explanation is that successful detection of precursor and product ions, and their subsequent DT alignment, can be interfered by coeluting ions with identical/similar DT. Thus, by utilizing alternative cIM routines, interference can be reduced by improved separation of the interfering ions in DT. Nevertheless, the unique peptide populations generated by combined one-pass/multipass peptide mapping can be integrated to generate larger reference peptide databases for use in subsequent HDX-MS experiments.

### Evaluating Novel HDX-cIM-MS Workflow under Deuterated Conditions

**Figure 5:**
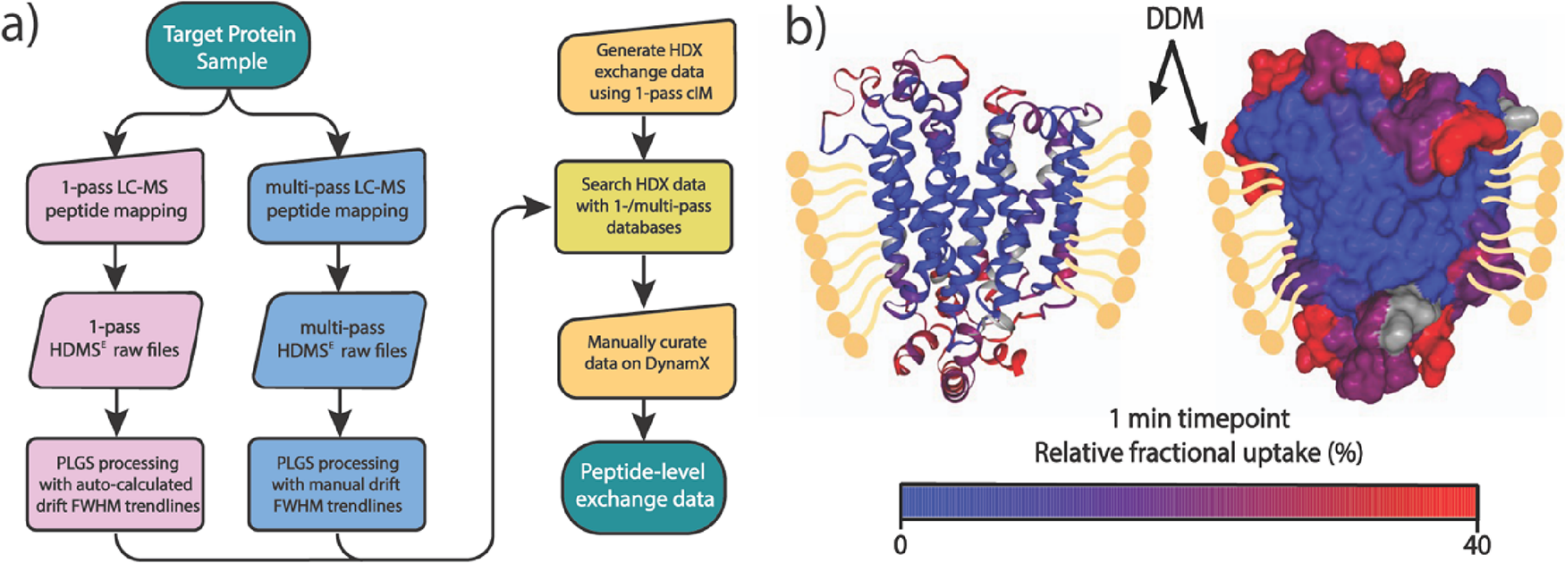
(a) Flowchart outlining novel HDX-MS workflow with combined one-pass/multipass peptide mapping. (b) Homology models of WT XylE (based on 4GBY^47^) in cartoon and surface format. One-minute HDX data is heat mapped onto structures as relative fractional uptake, with unsequenced regions in gray.

Owing to the increased complexity of deuterated peptide isotopic distributions, it is typical for many peptides to be excluded during HDX-MS analysis. Therefore, it is important to manually validate peptides under deuterated conditions to ensure they are suitable for subsequent HDX-MS. To do this, both one-pass and multipass reference peptide databases were used to search for deuterated peptides in a 60 s HDX time point acquired using either standalone one-pass (SMO, MsbA, and SecYEG) or multipass (XylE) cIM. The data was then analyzed using reference peptide databases generated using one-pass cIM alone for comparison (Figure 5a).

Under deuterated conditions, 93% of all peptides from combined one-pass/multipass peptide mapping were retained in HDX-MS readout. Moreover, combined one-pass/multipass peptide mapping provided successful exchange monitoring of 220 additional peptides across all proteins relative to one-pass or multipass alone (Table S2). Thus, this novel approach not only provides increased peptide identification but also provides high-quality peptides, which are monitorable in subsequent HDX-MS experiments. This is supported by the strong correlation between the observed HDX pattern and known IMP transmembrane topology (Figure 5b and Figures S8–17). For XylE and SMO, combined one-pass/multipass peptide mapping provided HDX-MS coverages of 88 and 95%, respectively, while this dropped to 85 and 89% when using one-pass alone. XylE and SMO also exhibited 30 and 31% higher redundancy, respectively. Thus, while the additional peptide identifications appear to favor increased redundancy, they can also help retain HDX-MS coverage. For MsbA and SecYEG, no difference in HDX-MS coverage was observed between combined one-pass/multipass peptide mapping and one-pass cIM alone. However, for MsbA and SecYEG, combined one-pass/multipass provided 27 and 5% increases in redundancy, respectively (Table S2). Crucially, the ability to monitor peptides from combined one-pass/multipass peptide mapping, when using only a single cIM routine for the HDX time points, demonstrates that the vast majority of peptides that are unique to one cIM routine are monitorable in subsequent HDX-MS experiments using the opposing cIM routine. Consequently, there appears to be limited benefit to using both one-pass and multipass throughout HDX time points. Therefore, we recommend utilizing both one-pass and multipass cIM routines during peptide mapping but using a single cIM routine throughout HDX time points to minimize the number of required time point replicates.

### Conclusions and Future Outlook

Here, we propose a novel HDX-MS workflow with integrated cIM that has potential to help generate higher-quality HDX-MS data from more challenging targets in the future. Benchmarking against previous generation SYNAPT G2-Si instrumentation demonstrated the capability of cyclic IMS to improve bottom-up LC-MS data quality under identical experimental conditions. Its increased sensitivity, dynamic range, and mobility resolution enabled an increase in sequence coverage or redundancy for all assessed targets.

Counterintuitively, use of multipass cIM initially resulted in underperformance relative to one pass. Manual inspection of multipass data revealed the presence of high-quality peptide ions not captured by default PLGS processing. This is likely due to the wrap-around-derived loss of correlation between DT and drift FWHM, which, in turn, prevents Apex3D from applying appropriate smoothing kernels. To address this, we developed a novel HDMS^E^ data processing approach that uses user-defined drift FWHM trendlines for more appropriate smoothing of multipass cIM data. This led to substantial increases in peptide identification when using multipass, with some examples exhibiting data quality comparable to one pass. While the 11–14 mobility peak width trendline improved peptide identification across all targets in this study, it is possible that this trendline is not optimal to all targets and/or multipass cIM routines. Thus, we recommend optimization of drift FWHM trendlines for new targets and/or multipass cIM sequences, as exemplified in this manuscript.

It has previously been demonstrated that multipass data can be “unwrapped” using zero-pass, one-pass, and multipass cIM in conjunction to calculate the periodic DT.^44^ Utilization of this technique in HDMS^E^ workflows would be beneficial, as the derived periodic DT could once again by used to autocalculate trendlines, which correlate drift FWHM with periodic DT. Ideally, instruments would dynamically switch between different cIM routines throughout HDMS^E^ experiments, in a manner analogous to low-energy/high-energy switching, to provide zero-pass, one-pass and multipass data simultaneously.

The use of one-pass and multipass cIM routines generated unique peptide populations, which were combined to improve overall data quality. Consequently, we recommend performing both one-pass and multipass peptide mapping prior to HDX-MS analyses to fully exploit instrument performance and bolster nondeuterated peptide databases. This approach would also benefit from dynamic switching between different cIM routines throughout HDMS^E^, as both one-pass and multipass data could be acquired simultaneously to increase analytical throughput.

While combined one-pass/multipass peptide mapping provided the highest number of peptides identified for all assessed targets, there remained some sequence segments that were not captured (e.g., TMD3 of SMO). Thus, despite use of combined one-pass/multipass cIM peptide mapping, there are further gains to be achieved. Increased peak capacity via cIM in combination with other strategies, such as optimized CE ramps,^11^ alternative proteases,^48^ optimized quench buffer compositions,^12^ and subzero chromatography,^13,14,49,50^ is likely the route to deriving optimal sequence coverage for challenging targets such as IMPs in future. Nevertheless, the HDX-MS workflow proposed in this manuscript still holds great potential to improve HDX-MS analyses for any target protein with both previously optimized and nonoptimized experimental conditions. It is important to note that the combined one-pass/multipass approach taken herein is not only applicable to HDX-MS; it is applicable to any bottom-up LC-MS and omics applications, which use LC-MS/MS for the identification of molecules in complex mixtures. Thus, we envision that widespread adoption of this approach has the potential to improve analyses in adjacent fields in the future.

## Data Availability

The mass spectrometry proteomics data has been deposited to the ProteomeXchange Consortium via the PRIDE^51^ partner repository with the data set identifier PXD048293. Other data are available upon reasonable request.

## Supporting Information Available

MsbA expression and purification; XylE expression and purification; SecYEG expression and purification; SMO (BRIL) expression and purification; LC instrumentation and conditions; sample injection gradient table; sawtooth wash gradient table; SELECT SERIES Cyclic IMS instrument tuning parameters; cyclic one-pass mobility tuning parameters; cyclic multipass mobility tuning parameters; SYNAPT G2-Si instrument tuning parameters; Apex3D/Peptide3D processing via windows command prompt instructions; benchmarking Cyclic IMS performance relative to SYNAPT G2-Si (Figure S1); manual comparison of SYNAPT G2-Si and Cyclic IMS raw data (Figure S2); drift time vs drift FWHM for SMO, MsbA, and SecYEG (Figure S3); Driftscope display (drift time vs *m*/*z*) for XylE (Figure S4); comparative coverage maps for one-pass/multipass (11–14) peptide mapping (Figure S5); number of peptide identifications for SYNAPT G2-Si vs cyclic one-pass vs cyclic combined one-pass/multipass (Figure S6); one-pass and multipass peptide threshold parameters comparison (Figure S7); XylE one-pass HDX-MS heat map (Figure S8); XylE multipass HDX-MS heat map (Figure S9); SMO one-pass HDX-MS heat map (Figure S10); SMO multipass HDX-MS heat map (Figure S11); MsbA one-pass HDX-MS heat map (Figure S12); MsbA multipass HDX-MS heat map (Figure S13); SecY one-pass HDX-MS heat map (Figure S14); SecY multipass HDX-MS heat map (Figure S15); SecE one-pass and multipass HDX-MS heat maps (Figure S16); SecG one-pass and multipass HDX-MS heat maps (Figure S17); SYNAPT G2-Si vs Cyclic IMS peptide mapping overview (Table S1); and cyclic one-pass vs cyclic one-pass/multipass HDX-MS overview (Table S2) (PDF)
