# Supplementary material for: Cyclic Ion Mobility for Hydrogen/Deuterium Exchange-Mass Spectrometry Applications
Source: Anal Chem. 2024 Apr 1;96(15):5869–77. doi: 10.1021/acs.analchem.3c05753 (PMC11024883; doi:10.1021/acs.analchem.3c05753)
Supplement: Supplementary file 1 — ac3c05753_si_001.pdf [file ac3c05753_si_001.pdf]

# Supporting Information

# Cyclic ion mobility for hydrogen/deuterium exchange-mass spectrometry applications

Damon Griffiths[1,2], Malcolm Anderson[3], Keith Richardson[3], Satomi Inaba-Inoue[4,5,6], William J. Allen[7], Ian Collinson[7], Konstantinos Beis[4,5], Michael Morris[3], Kevin Giles[3], Argyris Politis [1,2,*]

[1] Faculty of Biology, Medicine and Health, School of Biological Sciences, The University of Manchester, Manchester M13 9PT, UK.

[2] Manchester Institute of Biotechnology, University of Manchester, Princess Street, Manchester, M1 7DN, UK.

[3] Waters Corporation, Stamford Avenue, Altrincham Road, Wilmslow SK9 4AX, UK.

[4] Department of Life Sciences, Imperial College London, Exhibition Road, South Kensington, London SW7 2AZ, UK.

[5] Rutherford Appleton Laboratory, Research Complex at Harwell, Didcot, Oxfordshire OX11 0FA, UK.

[6] Diffraction and Scattering Division, Japan Synchrotron Radiation Research Institute, SPring-8, 1-1-1, Kouto, Sayo, Hyogo 679-5198, Japan.

[7] School of Biochemistry, University of Bristol, Bristol BS8 1TD, UK

*Correspondence: argyris.politis@manchester.ac.uk

# Table of Contents


**MsbA expression and purification** ........ **S3**
**XylE expression and purification** ........ **S3**
**SecYEG expression and purification.** ........ **S3**
**SMO (BRIL) expression and purification.** ........ **S3**
**LC instrumentation and conditions** ........ **S4**
**Sample Injection gradient table** ........ **S5**
**Saw-tooth wash gradient table** ........ **S5**
**SELECT SERIES Cyclic IMS instrument tuning parameters** ........ **S6**
**Cyclic 1-pass mobility tuning parameters** ........ **S6**
**Cyclic multi-pass mobility tuning parameters** ........ **S7**
**SYNAPT G2-Si instrument tuning parameters** ........ **S7**
**Apex3D/Peptide3D processing via Window command prompt instructions** ........ **S8**
***Figure S1 - Benchmarking Cyclic IMS performance relative to SYNAPT G2-Si*** ........ ***S10***
***Figure S2 – Manual comparison of SYNAPT G2-Si and Cyclic IMS raw data.*** ........ ***S11***
***Figure S3 - Drift time vs Drift FWHM for SMO, MsbA and SecYEG.*** ........ ***S12***
***Figure S4 – Driftscope display (Drift time vs m/z) for XylE.*** ........ ***S13***
***Figure S5 – Comparative coverage maps of 1-pass/multi-pass (11-14) peptide mapping*** ........ ***S14***
***Figure S6 –Number of peptide identifications for SYNAPT G2-Si Vs Cyclic 1-pass vs Cyclic combined 1-pass/multi-pass.*** ........ ***S15***
***Figure S7 - 1-pass and multi-pass peptide threshold parameter comparison.*** ***S16***
***Figure S8 – XylE 1-pass HDX-MS heat map.*** ........ ***S17***
***Figure S9 – XylE multi-pass HDX-MS heat map*** ........ ***S18***
***Figure S10 – SMO 1-pass HDX-MS heat map.*** ........ ***S19***
***Figure S11 – SMO multi-pass HDX-MS heat map*** ........ ***S20***
***Figure S12 – MsbA 1-pass HDX-MS heat map.*** ........ ***S21***
***Figure S13 – MsbA multi-pass HDX-MS heat map.*** ........ ***S22***
***Figure S14 – SecY 1-pass HDX-MS heat map.*** ........ ***S23***
***Figure S15 – SecY multi-pass HDX-MS heat map.*** ........ ***S24***
***Figure S16 – SecE 1-pass and multi-pass HDX-MS heat maps*** ........ ***S25***
***Figure S17 – SecG 1-pass and multi-pass HDX-MS heat maps.*** ........ ***S26***
***Table S1 – SYNAPT G2-Si vs Cyclic IMS peptide mapping overview.*** ........ ***S27***
***Table S2 – Cyclic 1-pass vs Cyclic 1-pass/multi-pass HDX-MS overview.*** ........ ***S27***

***Supporting Information References*......................................................................***S28***

**MsbA expression and purification.** The *msbA* gene was subcloned into a pET-28b vector containing a C-terminal $His_{10}$-tag and a Tobacco Etch Virus (TEV) protease site. The plasmid was transformed into BL21 (DE3) cells. Cells were grown in Luria Bertani media containing 50 µg/ml kanamycin. Cells were induced with 0.5 mM IPTG overnight at 25 °C. Purification was carried out as previously described. [1] MsbA was purified to homogeneity as judged by SDS-PAGE. The final buffer after size exclusion chromatography was 20 mM Tris HCl pH 7.4, 150 mM NaCl, 0.02% facade®-EM.

**XylE expression and purification.** XylE expression and purification was carried out as previously described. [2]

**SecYEG expression and purification**. SecYEG expression and purification was carried out as previously described. [3]

**SMO (BRIL) expression and purification.** The SMO construct used was a human SMO with an ICD truncation and BRIL fusion protein replacing ICL3. pEG BacMam plasmid vectors were cloned with an N-terminal FLAG/Twin-Strep tag. Using a baculovirus-mediated transfection system, SMO constructs were expressed in HEK293S cells in FreeStyle™ media (supplemented with 3.75 mM valproic acid) with a multiplicity of infection (MOI) of 1. Cells were then placed in a humidified incubator (125 r.p.m, 37°C and 8% $CO_2$ for 24 hours) for protein expression. Once expressed, cells were centrifuged (3200 g for 3 min at 4°C) and pellet resuspended in phosphate buffered saline (PBS) supplemented with protease inhibitor, followed by centrifugation (3200 g for 3 min at 4°C) and resuspension in solubilisation buffer (50 mM HEPES, 200 mM NaCl, 1 protease inhibitor tablet, pH 7.5). Detergent stock (DDM/CHS;

10%/1%) was immediately added to a final concentration of 1.5% and left to solubilise on agitator (7 r.p.m at 4°C for 1 hour). After solubilisation, the supernatant was collected via centrifugation (25,000 g for 30 mins at 4°C), followed by addition of streptavidin sepharose resin which was left to bind to protein on an orbital roller (5 r.p.m at 4°C for 2 hours). Once bound, protein-strep resin samples were centrifuged (100 g for 3 min at 4°C) in a filtered centrifugal column and resuspended in wash buffer A (50mM HEPES (pH 7.5), 200mM NaCl, 0.03% DDM/CHS, 5mM ATP/$MgCl_2$). This process was repeated with 20 column volumes (CV) wash buffer A, followed by 10 CV with wash buffer B (50mM HEPES (pH 7.5), 200mM NaCl, 0.03% DDM/CHS), and finally 6 x 0.5 CV with elution buffer (50mM HEPES (pH 7.5), 200mM NaCl, 0.03% DDM/CHS, 2.5 mM desthiobiotin). PNGase was added to purified samples in a ratio of 1:20 (receptor:protease) and incubated at RT for 2 hours for deglycosylation. To remove PNGase, FLAG resin was added and left to bind on an orbital roller (5 r.p.m at 4°C for 2 hours). Once bound, protein-FLAG resin samples were centrifuged (100 g for 3 min at 4°C) and resuspended in 20 CV wash buffer A, followed by 10 CV wash buffer B and 6 x 0.5 CV elution buffer (50mM HEPES (pH 7.5), 200mM NaCl, 0.03% DDM/CHS, 0.1 mg/mL FLAG peptide). All streptavidin and/or FLAG purified proteins were then applied to a Superose 6 10/300 gel filtration column equilibrated with SEC buffer (50mM HEPES, 200mM NaCl, 0.03% DDM/CHS)

## LC instrumentation and conditions

| | |
|---|---|
| **LC System** | ACQUITY UPLC M Class with HDX manager |
| **Robotics** | Trajan LEAP HDX automation platform |
| **Vials** | Waters Total recovery |
| **Digestion column** | BEH Enzymate Pepsin column |
| **Trap column** | ACQUITY UPLC 2.1 x 5 mm BEH $C_{18}$ VanGuard pre-column |

| | |
|---|---|
| **Analytical column** | ACQUITY UPLC 1 x 100 mm BEH $C_{18}$ Column, 1.7 μm |
| **Digestion column temperature** | 20°C |
| **HDX manager temperature** | 1°C |
| **Injection volume** | 50 μL |
| **Auxiliary solvent manager (ASM) flow rate** | 100 μL/min |
| **Binary solvent manager (BSM) flow rate** | 40 μL/min |
| **Mobile phase A** | 0.2% formic acid in $H_20$ |
| **Mobile phase B** | 0.2% formic acid in acetonitrile |
| **Pepsin washes per sample injection** | 1 |
| **Pepsin wash composition** | 1.5 M Gu-HCl, 0.4% MeOH, 0.5% formic acid, pH 3 |

## Sample Injection gradient table

| Time (min) | Flow rate (μL/min) | %A | %B |
|---|---|---|---|
| **Trapping: 0.00** | 100 | 100 | NA |
| **Trapping: 3.00** | 100 | 100 | NA |
| **Analytical: 0.00** | 40 | 92 | 8 |
| **Analytical: 8.00** | 40 | 45 | 55 |
| **Analytical: 8.50** | 40 | 15 | 85 |
| **Analytical: 9.50** | 40 | 15 | 85 |
| **Analytical: 10.00** | 40 | 92 | 8 |
| **Analytical: 13.00** | 40 | 92 | 8 |

## Saw-tooth wash gradient table

| Time (min) | Flow rate (μL/min) | %A | %B |
|---|---|---|---|
| **Trapping: 0.00** | 100 | 100 | NA |
| **Trapping: 3.00** | 100 | 100 | NA |
| **Analytical: 0.00** | 40 | 92 | 8 |
| **Analytical: 2.00** | 40 | 10 | 90 |

| | | | |
|---|---|---|---|
| **Analytical: 4.00** | 40 | 10 | 90 |
| **Analytical: 6.00** | 40 | 92 | 8 |
| **Analytical: 8.00** | 40 | 92 | 8 |
| **Analytical: 10.00** | 40 | 10 | 90 |
| **Analytical: 12.00** | 40 | 10 | 90 |
| **Analytical: 14.00** | 40 | 92 | 8 |

## SELECT SERIES Cyclic IMS instrument tuning parameters

| | |
|---|---|
| **Ionisation Polarity** | Positive |
| **Acquisition mode** | V-mode $HDMS^E$ |
| **Acquisition range (m/z)** | 50-2000 |
| **Capillary voltage (kV)** | 2.0 |
| **Cone gas flow (L/hr)** | 0 |
| **Desolvation gas flow (L/hr)** | 800 |
| **Desolvation temperature (°C)** | 550 |
| **Nebulizer gas flow (bar)** | 6 |
| **Source temperature (°C)** | 100 |
| **Sampling cone voltage (V)** | 30 |
| **Fragmentation mode** | CID |
| **Scan time** | 0.3 |
| **Transfer collision energy ramp (V)** | 15-50 |
| **Quad profile (MS Mass 1 / 2 / 3)** | 400 / 500 / 600 |

## Cyclic 1-pass mobility tuning parameters

| | |
|---|---|
| **Dead time offset (V)** | 1.50 |
| **Entry offset (V)** | 5 |
| **Exit offset (V)** | 2 |
| **Gate TW height (V)** | 6 |
| **Gate TW velocity (m/s)** | 375 |

| | |
|---|---|
| **Racetrack TW height (V)** | 23 |
| **Racetrack TW Velocity (m/s)** | 375 |
| **Pushes per bin** | 2 |
| **ADC delay (ms)** | 13 |
| **Injection sequence (ms)** | 10 |
| **Separation sequence (ms)** | 3 |
| **Ejection/acquire sequence (ms)** | 34 |
| **Scan time after adjustment** | 0.3336 |

## Cyclic multi-pass mobility tuning parameters

| | |
|---|---|
| **Dead time offset (V)** | 1.50 |
| **Entry offset (V)** | 5 |
| **Exit offset (V)** | 2 |
| **Gate TW height (V)** | 6 |
| **Gate TW velocity (m/s)** | 375 |
| **Racetrack TW height (V)** | 22 |
| **Racetrack TW Velocity (m/s)** | 375 |
| **Pushes per bin** | 2 |
| **ADC delay (ms)** | 28 |
| **Injection sequence (ms)** | 10 |
| **Separation sequence (ms)** | 18.13 |
| **Ejection/acquire sequence (ms)** | 34 |
| **Scan time after adjustment** | 0.3139 |

## SYNAPT G2-Si instrument tuning parameters

| | |
|---|---|
| **Ionisation Polarity** | Positive |
| **Acquisition mode** | V-mode HDMS$^E$ |
| **Acquisition range (m/z)** | 50-2000 |
| **Capillary voltage (kV)** | 3.0 |

| | |
|---|---|
| **Cone gas flow (L/hr)** | 0 |
| **Desolvation gas flow (L/hr)** | 600 |
| **Desolvation temperature (°C)** | 550 |
| **Nebulizer gas flow (bar)** | 6.5 |
| **Source temperature (°C)** | 100 |
| **Sampling cone voltage (V)** | 30 |
| **Fragmentation mode** | CID |
| **Scan time** | 0.3336 |
| **Transfer collision energy ramp (V)** | 20-45 |
| **Quad profile (MS Mass 1 / 2 / 3)** | 400 / 500 / 600 |

**Apex3D/Peptide3D processing via Window command prompt instructions**

Download a batch file editor (e.g, notepad++ is a free software package that can create and edit batch files).

Copy and paste the following text into notepad++ and save as a new .bat file…

**For default processing:**

```
"C:\PLGS3.0.2\lib\apex3d\Apex3d64.exe" -pRawDirName "_" -outputDirName "_" -
outputUserDirName "_" -lockMassZ1 556.2766 -leThresholdCounts 250 -
heThresholdCounts 100 -lockMassToleranceAMU 0.3 -startingRTMin 2.0 -
endingRTMin 10 -PLGS
```

**If you want to specify a specific drift FWHM trendline:**

```
"C:\PLGS3.0.2\lib\apex3d\Apex3d64.exe" -pRawDirName "_" -outputDirName "_" -
outputUserDirName "_" -lockMassZ1 556.2766 -leThresholdCounts 250 -
heThresholdCounts 100 -lockMassToleranceAMU 0.3 -startingRTMin 2.0 -
endingRTMin 10 -driftFWHMStart 1 -driftFWHMEnd 4 -PLGS
```

Change the following where appropriate…

"C:\PLGS3.0.2\lib\apex3d\Apex3d64.exe": **The Apex3D64.exe file path location**
-pRawDirName: **The file path for the .raw file you wish to process**
-outputDirName: **File path you want the Apex3D processing output to go**
-outputUserDirName: **File path you want the Apex3D processing output to go**
-lockMassZ1: **m/z of lock mass**
-leThresholdCounts: **Low energy count threshold**
-heThresholdCount**: High energy count threshold**

-lockMassToleranceAMU: **Accepted lock mass tolerance range**
-startingRTMin: **The retention time processing will start at**
-endingRTMin: **The retention time processing will end at**
-driftFWHMStart: **The specified drift FWHM value at drift bin 1**
-driftFWHMEnd: **The specified drift FWHM value at drift bin 200**

Once saved, run the .bat file through the windows command prompt (This can be done by doubling clicking the .bat file in file explorer). This will process the specified .raw file via Apex3D and will produce an output .bin file in the specified output location.

For peptide3D processing, copy and paste the following text into notepad++ and save as a .bat file (this and all subsequent steps are identical for both default and specified drift FWHM processing.

“C:\PLGS3.0.2\lib\apex3d\Peptide3D.exe” -inputXMLFileName “_” -outputDirName “_” -outputUserDirName “_”

Change the following where appropriate…

“C:\PLGS3.0.2\lib\apex3d\Peptide3D.exe”: **The petide3D.exe file path location**
-inputXMLFileName: **The Petptide3D input file location (this will be the Apex3D BIN output)**
-outputDirName: **File path you want the Peptide3D processing output to go**
-outputUserDirName: **File path you want the Peptide3D processing output to go**

Peptide3D will process the Apex3D BIN file and produce an AMRT .xml file.

This .xml file can then be imported to PLGS by opening PLGS, right clicking on a well of the 96 well plate and clicking import spectrum. You can then process the data using typical workflow parameters that you would set during normal PLGS processing.

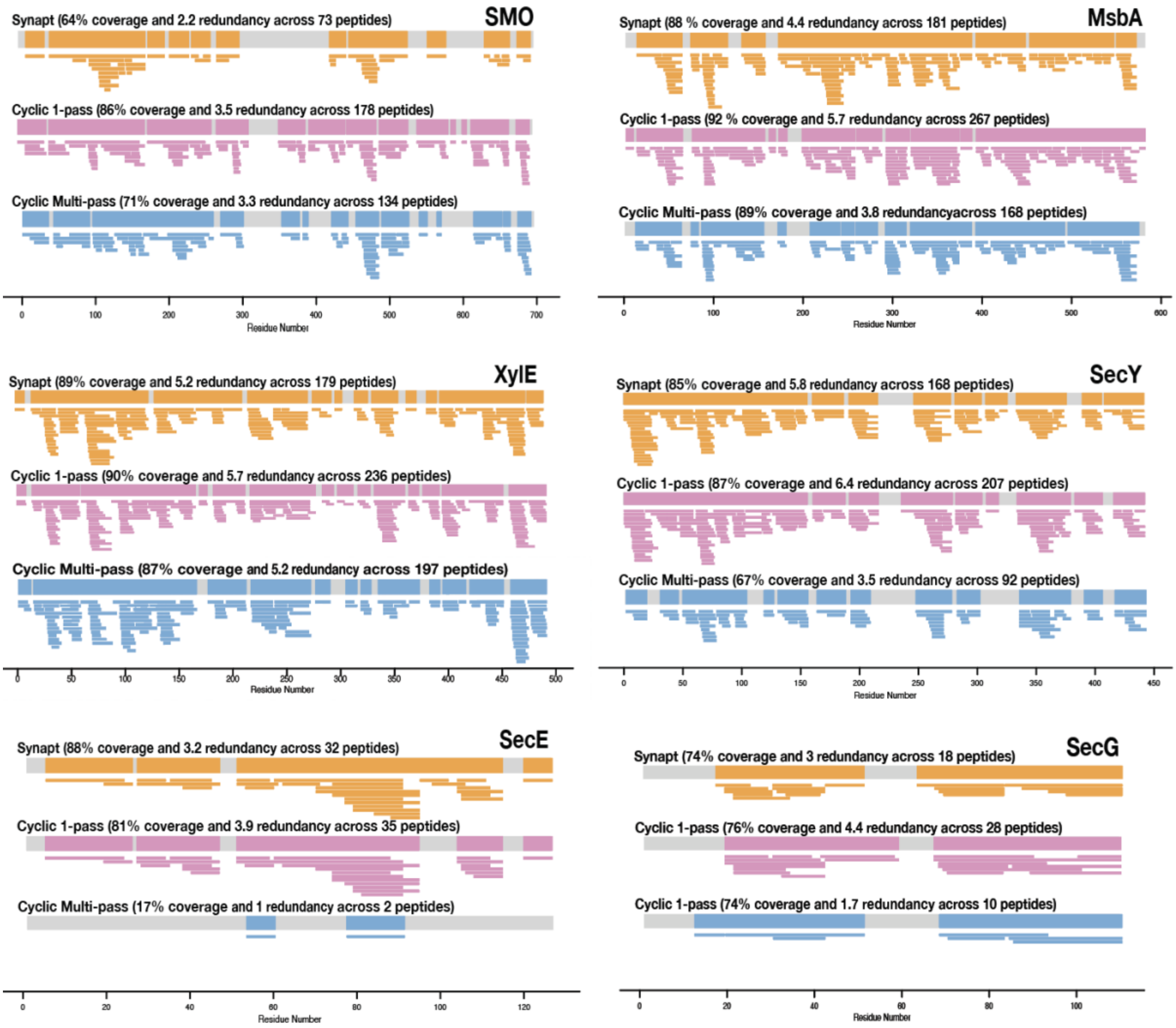


**Figure S1 - Benchmarking Cyclic IMS performance relative to SYNAPT G2-Si –** Sequence coverage maps for SMO (top left), XylE (middle left), SecE (bottom left), MsbA (top right), SecY (middle right), and SecG (bottom right) using SYNAPT G2-Si (orange), Cyclic IMS 1-pass (pink) and Cyclic IMS multi-pass (blue).

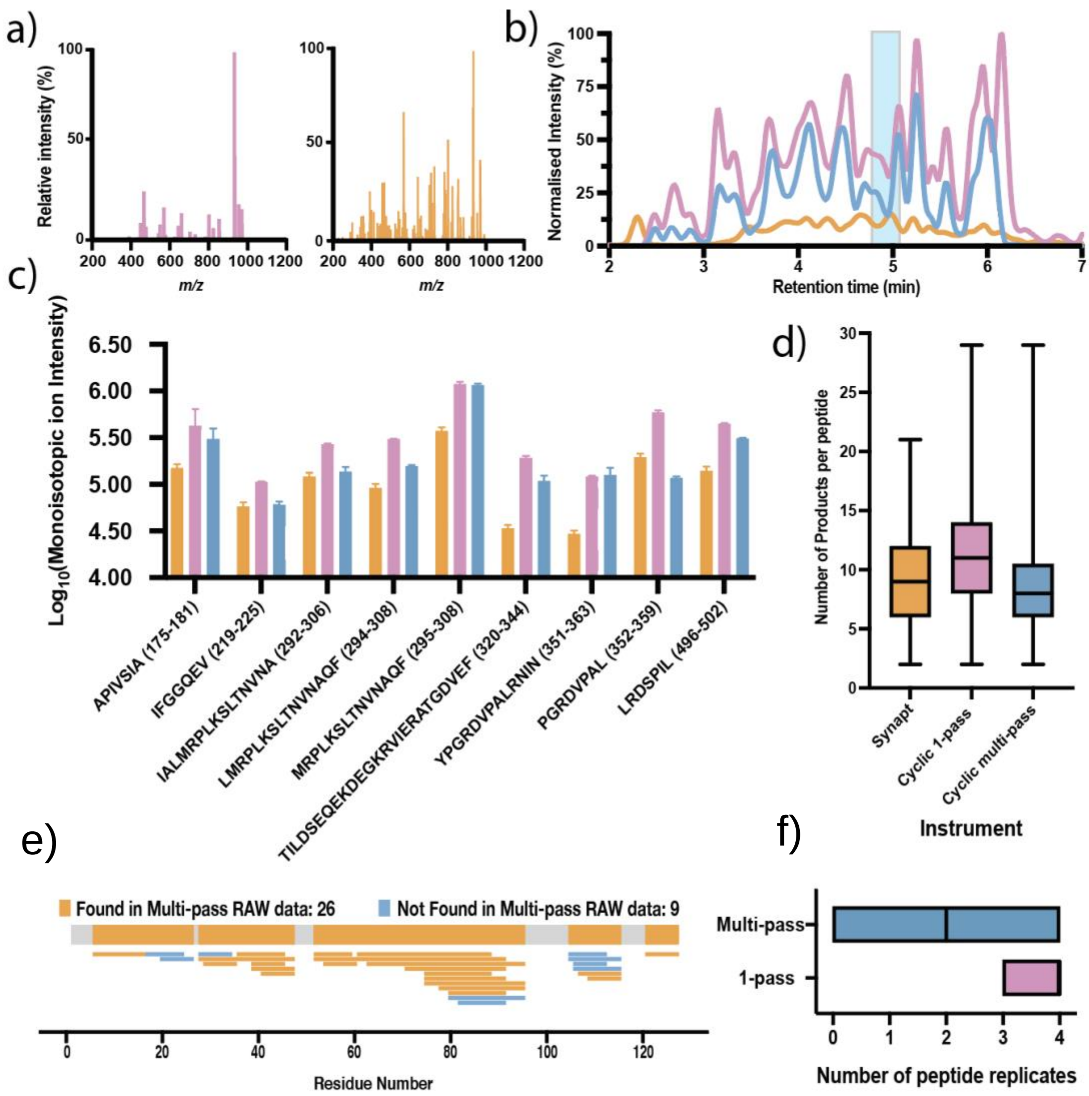


**Figure S2 – Manual comparison of SYNAPT G2-Si and Cyclic IMS raw data. (a)** Exemplar cyclic 1-pass (pink) and SYNAPT G2-Si (orange) MS spectra; taken from highlighted region in Figure S2b. Lower abundance ions are proportionally more intense in reference to base peak when using SYNAPT G2-Si owing to under-representation from detector saturation. **(b)** Typical BPI chromatograms of MsbA using SYNAPT G2-Si (orange), cyclic 1-pass (pink), and cyclic multi-pass (blue). **(c)** Log ion intensities for monoisotopic peak of common peptide ions in SYNAPT G2-Si (orange), cyclic 1-pass (blue) and cyclic multi-pass (pink) raw data. The spectra for this analysis was obtained on MassLynx by generating extracted ion chromatograms (EIC) form the monoisotopic peak of each ion, followed by generation of spectra by using >10% of total height of the EIC peak. Total intensities were then obtained then by automatic peak detection, followed by dividing total intensity by average single ion intensity (cyclic = 6.1, SYNAPT = 30). **(d)** Number of products per peptide for SMO, XylE, MsbA and SecYEG peptides which are common between SYNAPT GS-Si, cyclic IMS 1-pass and cyclic IMS multi-pass peptide mapping. **(e)** Comparative peptide coverage maps of SecE multi-pass and 1-pass raw data using SecE Cyclic 1-pass reference peptide database. Common peptides are in orange, whilst peptides only identified in cyclic 1-pass data are in blue. **(f)** Precursor ion identification across LC-MS replicates in cyclic 1-pass (mean of 3.91) and cyclic multi-pass (mean of 1.74) SecE data.

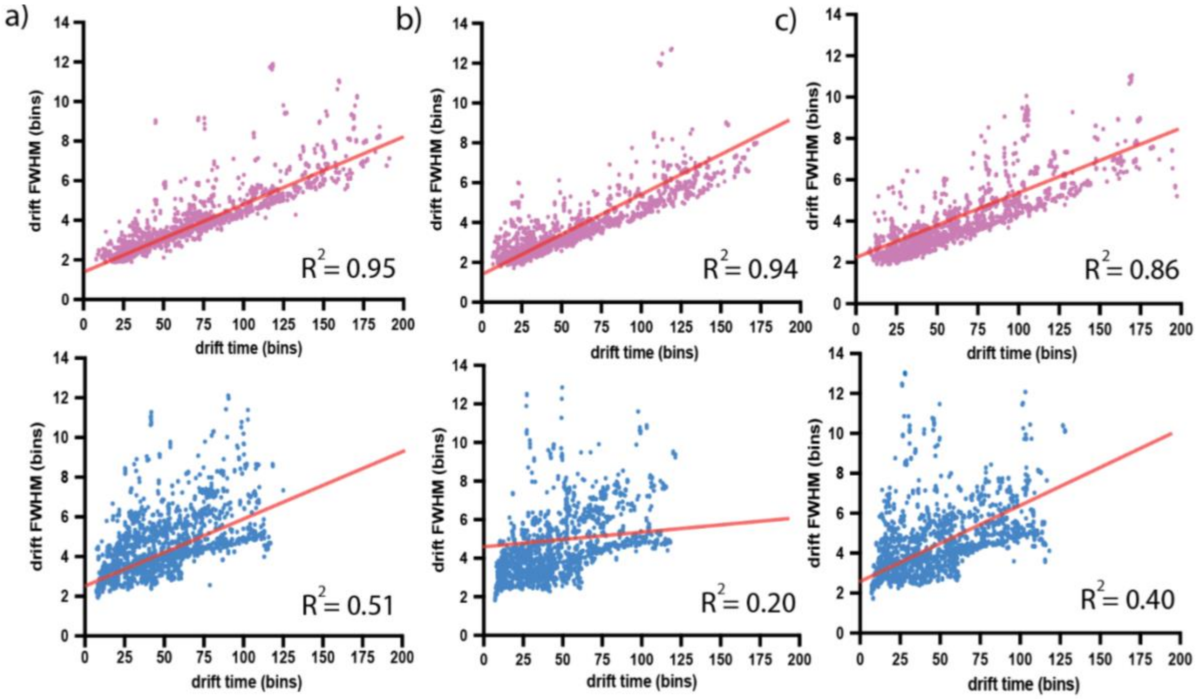


**Figure S3 - Drift time vs Drift FWHM for SMO, MsbA and SecYEG.** Apex3D calculated drift time vs drift FWHM values for top 2000 intense ions detected in SMO **(a),** MsbA **(b),** and SecYEG **(c)** peptide mapping data. Red lines illustrate drift FHWM trendlines auto calculated by Apex3D. Cyclic 1-pass experiments (top, pink) show strong positive correlation with high $R^2$ values. In contrast, cyclic multi-pass data (bottom, blue show drift FWHM trendlines with low positive correlation and $R^2$ values. Thus, a single compromise trendline is unlikely to appropriately smooth all IM data simultaneously.

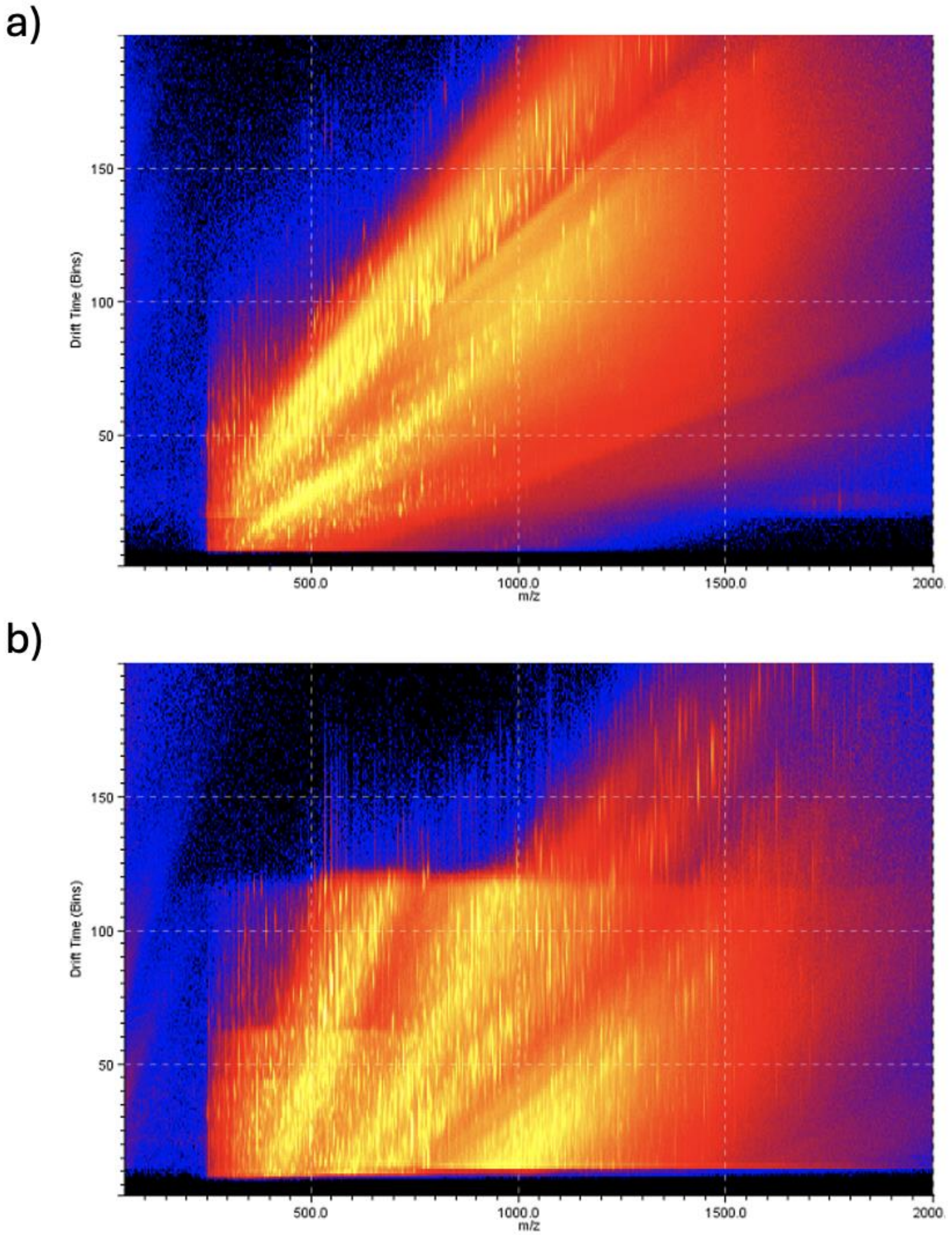


**Figure S4 – Driftscope display (Drift time vs m/z) for XylE.** (a) drift time vs m/z for ions detected by Driftscope ver 3.0 in XylE 1-pass data. (b) drift time vs m/z for ions detected by Driftscope ver 3.0 in XylE multi-pass data

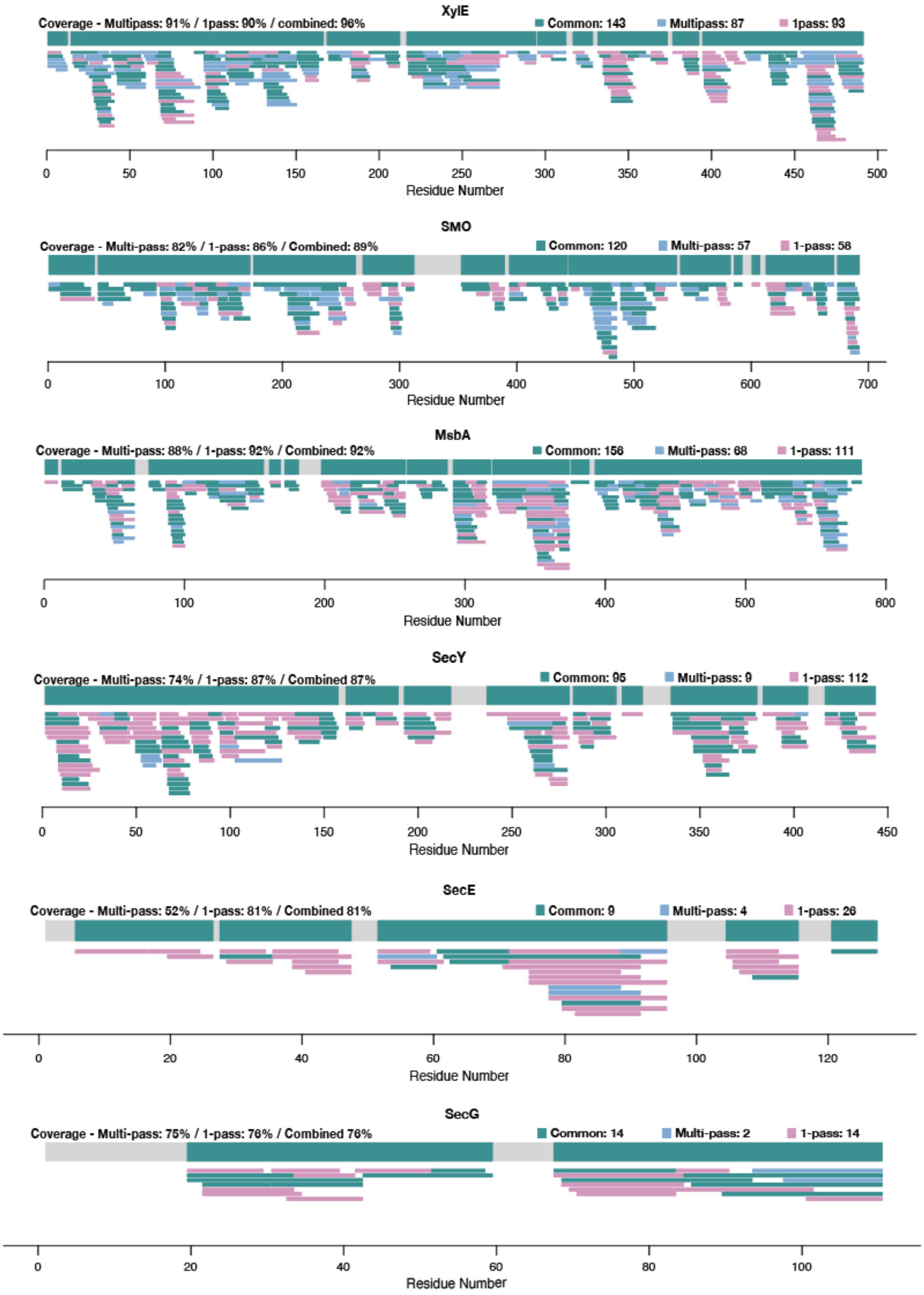


**Figure S5 – Comparative coverage maps of 1-pass/multi-pass (11-14) peptide mapping.** Comparative sequence coverage maps for XylE, SMO, MsbA, SecY, SecE, and SecG using 1-pass peptide mapping with auto-calculated drift FWHM trendline and multi-pass using manually inputted 11-14 drift FWHM trendline. Peptides unique to 1-pass are in pink, whilst peptides unique to multi-pass are in blue. Peptides common to both 1-pass and multi-pass are in green.

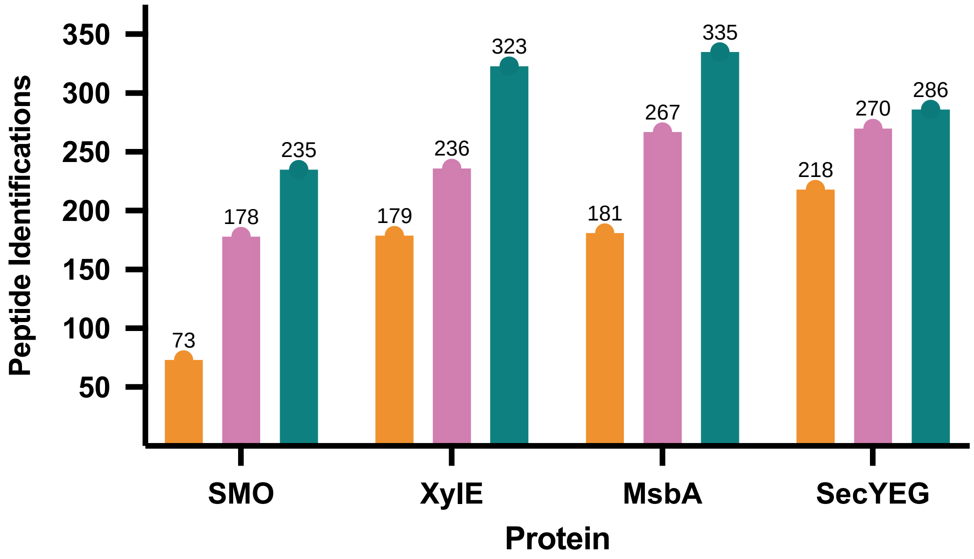


**Figure S6 –Number of peptide identifications for SYNAPT G2-Si Vs Cyclic 1-pass vs Cyclic combined 1-pass/multi-pass.** (**a**) Number of peptide identifications for SMO, XylE, MsbA, and SecYEG using SYNAPT G2-Si (orange) cyclic multi-pass with auto-calculated trendline (blue), cyclic 1-pass (pink), and the combined 1-pass/multi-pass peptide output (green).

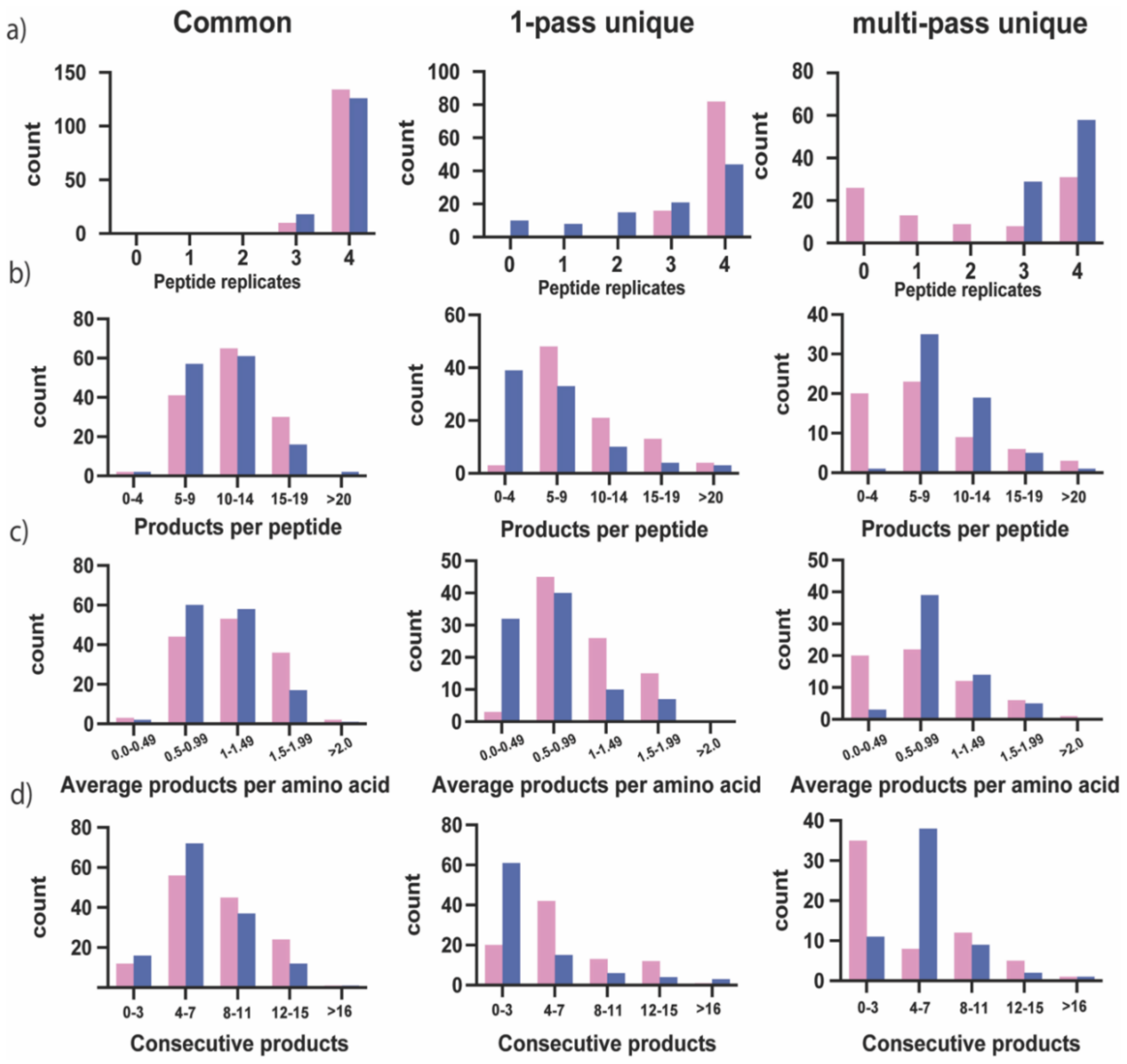


**Figure S7 - 1-pass and multi-pass peptide threshold parameter comparison.** Histogram frequency distributions for number of peptide replicates across LC-MS replicates (a), products per peptide (b), products per amino acid (c), and consecutively matched products (d) for the common, 1-pass unique and multi-pass unique peptide populations. Number of counts in the 1-pass raw PLGS database are shown in pink, while multi-pass (11-14) database counts are in dark blue. These values were generated by taking the curated common, 1-pass unique, and multi-pass unique peptide lists (generated after DynamX filtering and manual inspection), which were then used to extract corresponding peptide threshold parameter values from the raw 1-pass and multi-pass PLGS databases (only peptides that were present in at least 1 replicate of both 1-pass and multi-pass raw PLGS databases were included. For peptide identified in more than 1 replicate, the average values between replicates were used). These data sets were then split into back into the common, 1-pass unique and multi-pass unique sets (each column), and the threshold parameter values compared between the 1-pass (pink) and multi-pass (dark blue) raw PLGS databases. Thus, overall trends in peptide threshold values were compared between the raw 1-pass and multi-pass PLGS databases to assess why peptides were removed during DynamX filtering (i.e., common peptides show highly comparable frequency distributions when comparing 1-pass and multi-pass raw databases, while 1-pass unique and multi-pass unique peptides show significant downward shifts in frequency distribution of the opposing cIM routine).

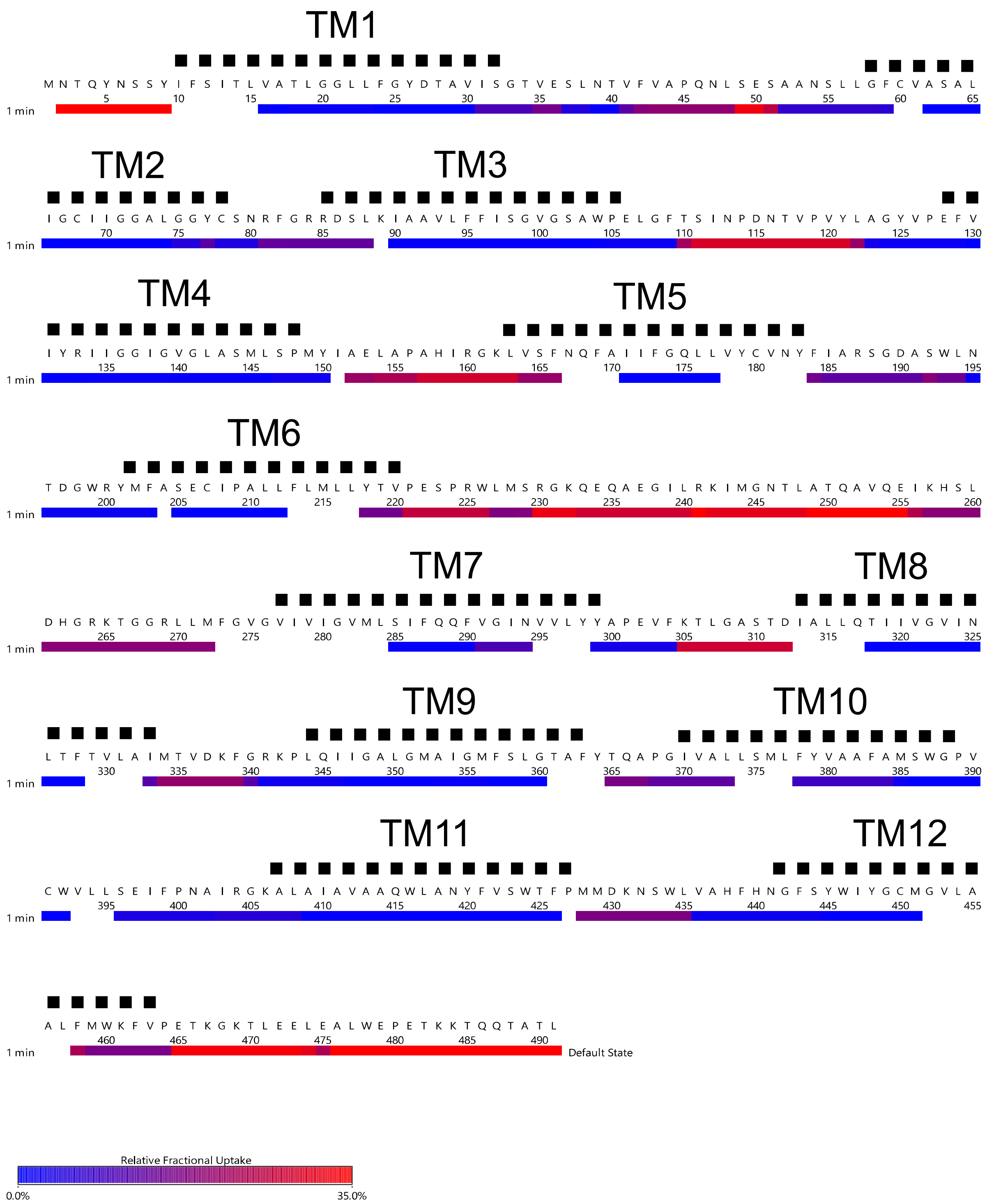


**Figure S8 – XylE 1-pass HDX-MS heat map.** Heat maps for 1min timepoint of XylE using cyclic 1-pass. Deuterium uptake is shown as relative fractional uptake, with the uptake of each residue being averaged across redundant peptides. Dashed black lines above the protein sequence represent known transmembrane domains/topology.

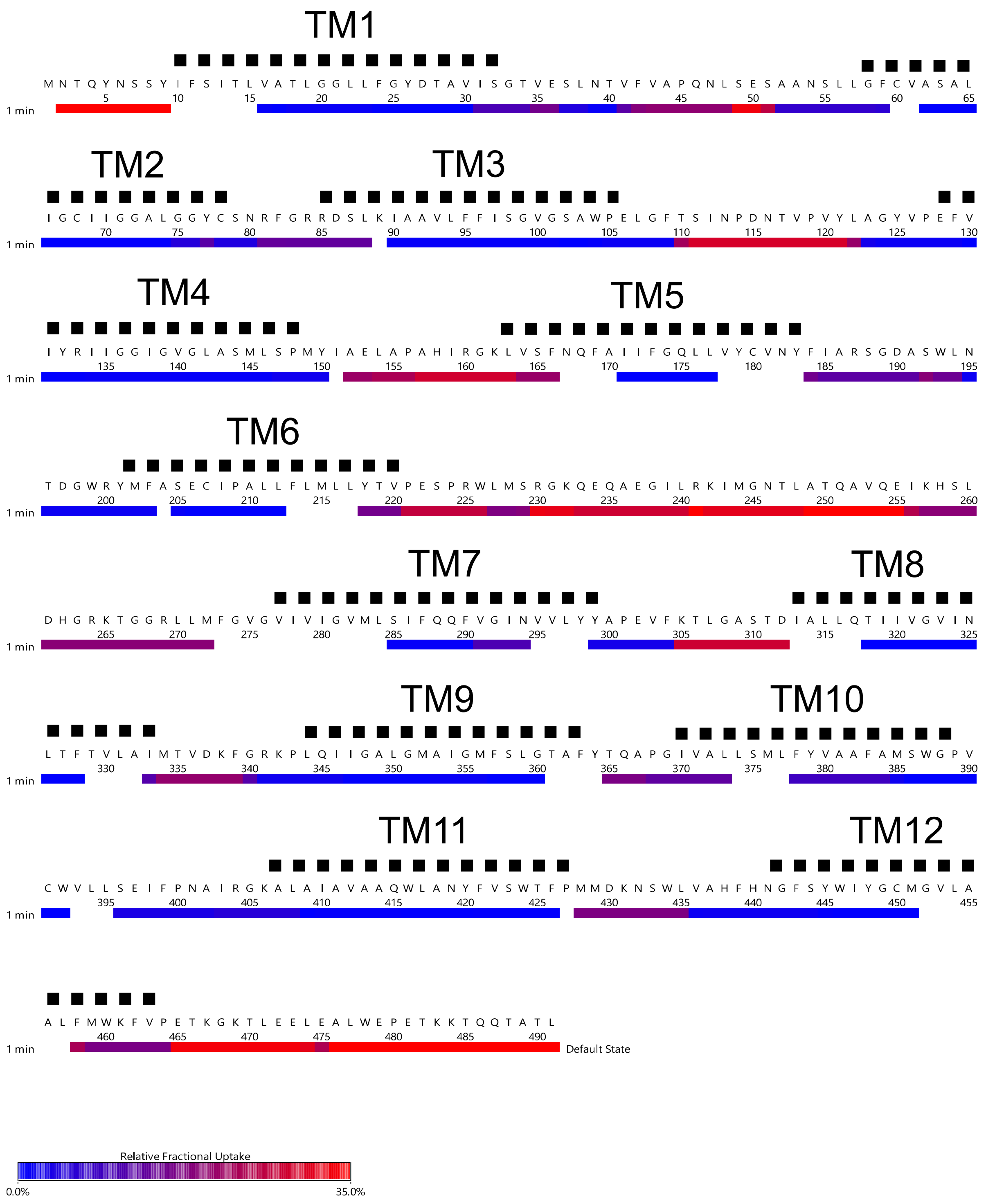


**Figure S9 – XylE multi-pass HDX-MS heat map.** Heat maps for 1min timepoint of XylE using cyclic multi-pass. Deuterium uptake is shown as relative fractional uptake, with the uptake of each residue being averaged across redundant peptides. Dashed black lines above the protein sequence represent known transmembrane domains/topology.

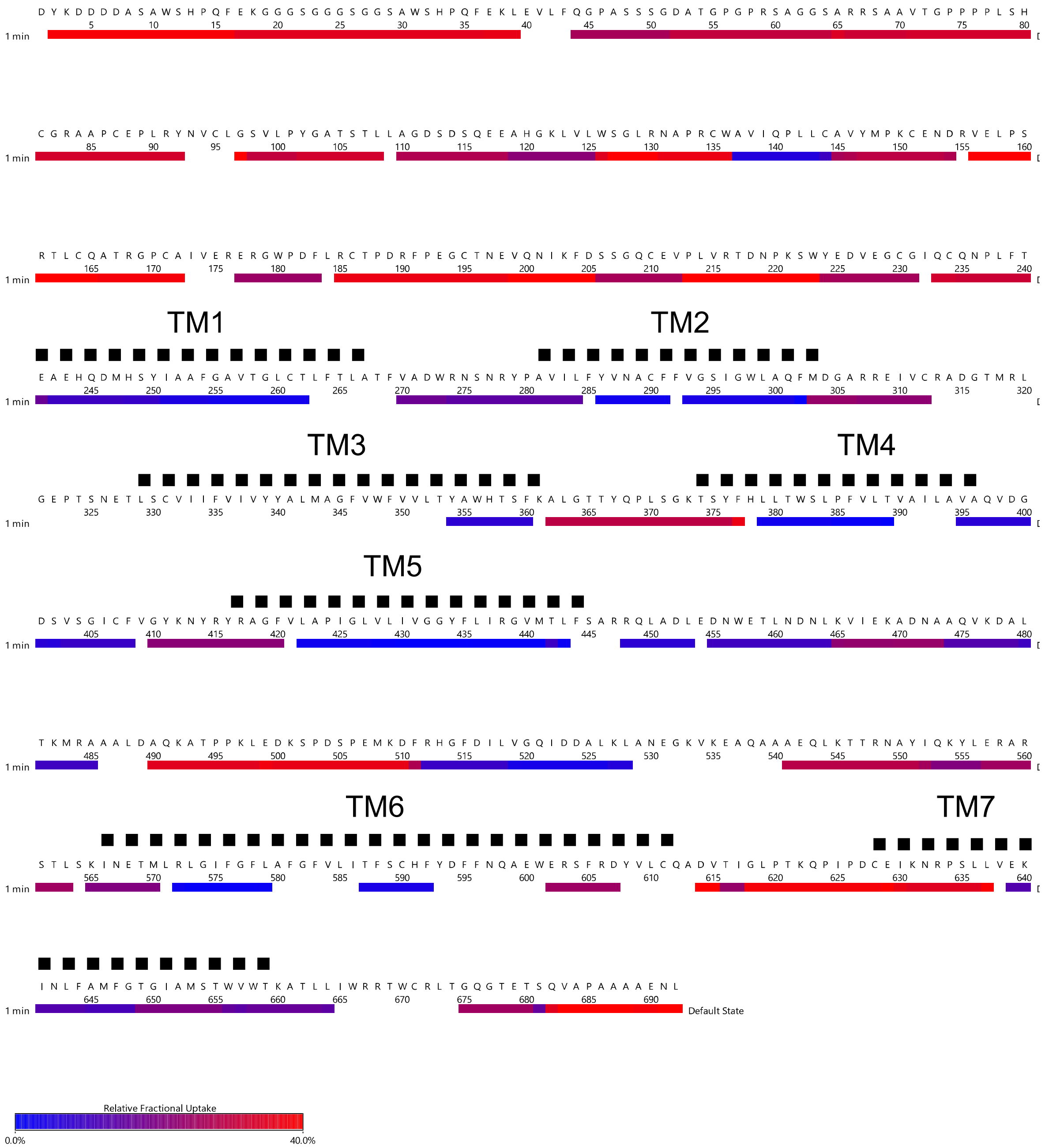

**Figure S10 – SMO 1-pass HDX-MS heat map**. Heat maps for 1min timepoint of SMO using cyclic 1-pass. Deuterium uptake is shown as relative fractional uptake, with the uptake of each residue being averaged across redundant peptides. Dashed black lines above the protein sequence represent known transmembrane domains/topology.

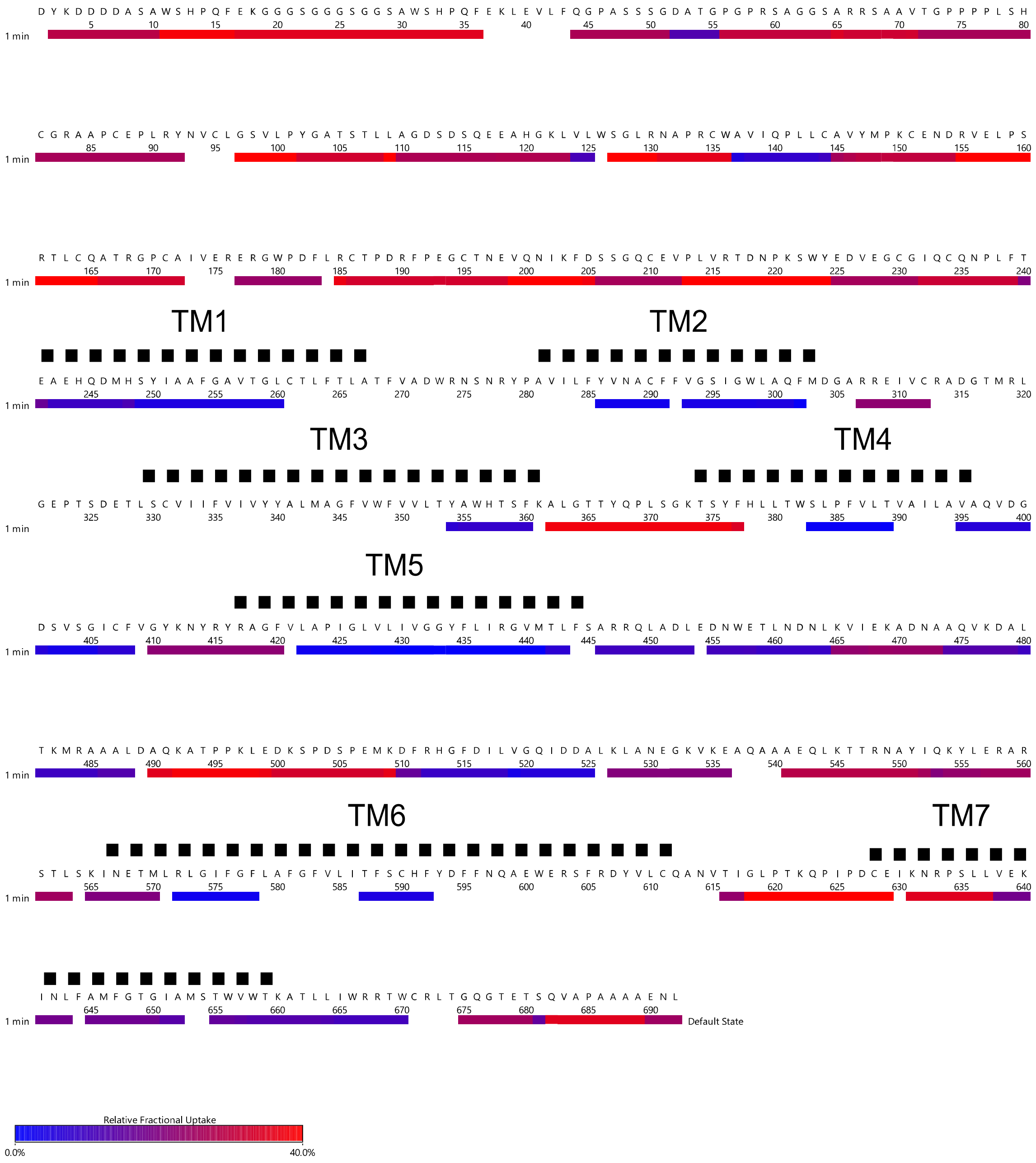


**Figure S11 – SMO multi-pass HDX-MS heat map.** Heat maps for 1min timepoint of SMO using cyclic multi-pass. Deuterium uptake is shown as relative fractional uptake, with the uptake of each residue being averaged across redundant peptides. Dashed black lines above the protein sequence represent known transmembrane domains/topology.

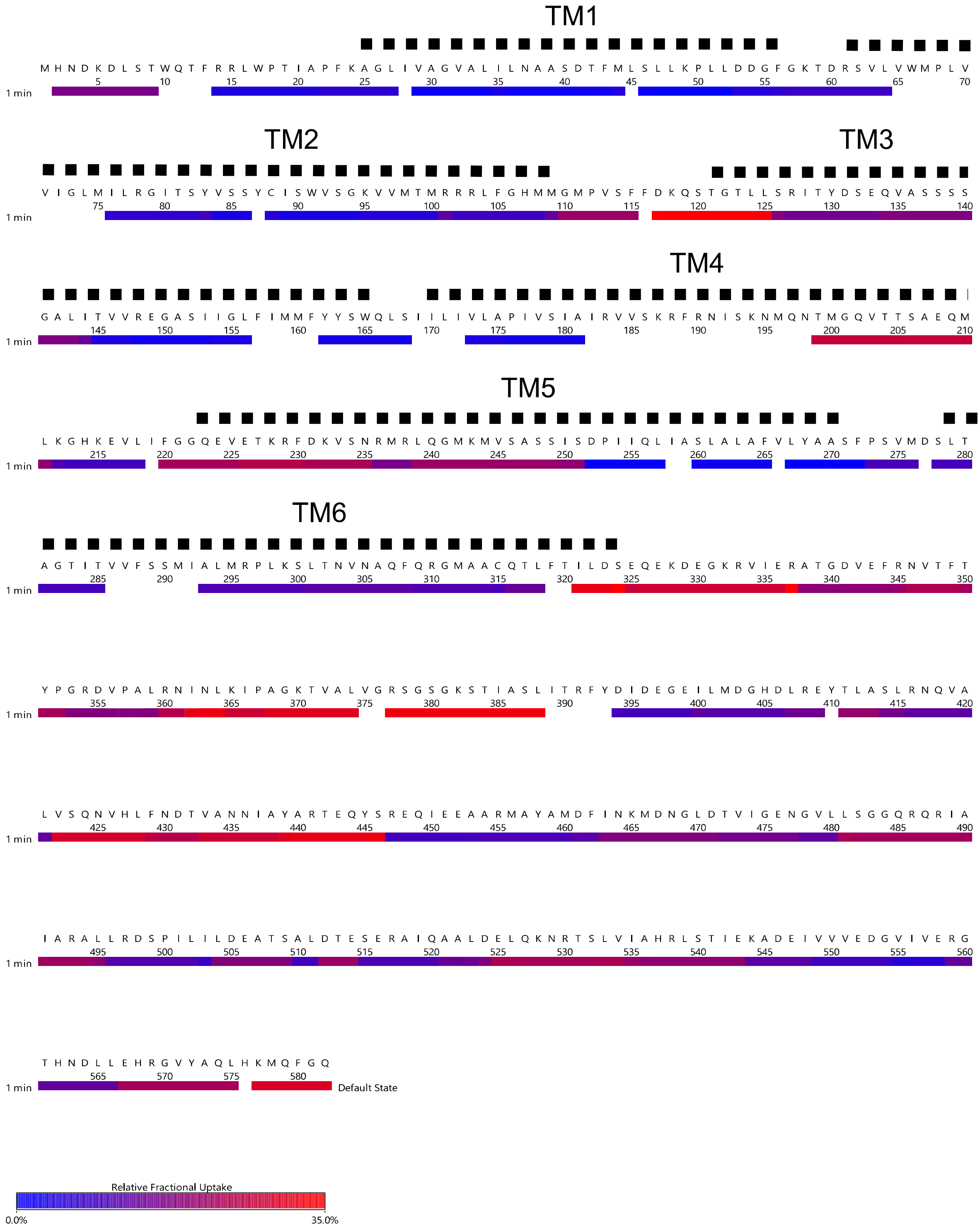

**Figure S12 – MsbA 1-pass HDX-MS heat map.** Heat maps for 1min timepoint of MsbA using cyclic 1-pass. Deuterium uptake is shown as relative fractional uptake, with the uptake of each residue being averaged across redundant peptides. Dashed black lines above the protein sequence represent known transmembrane domains/topology.

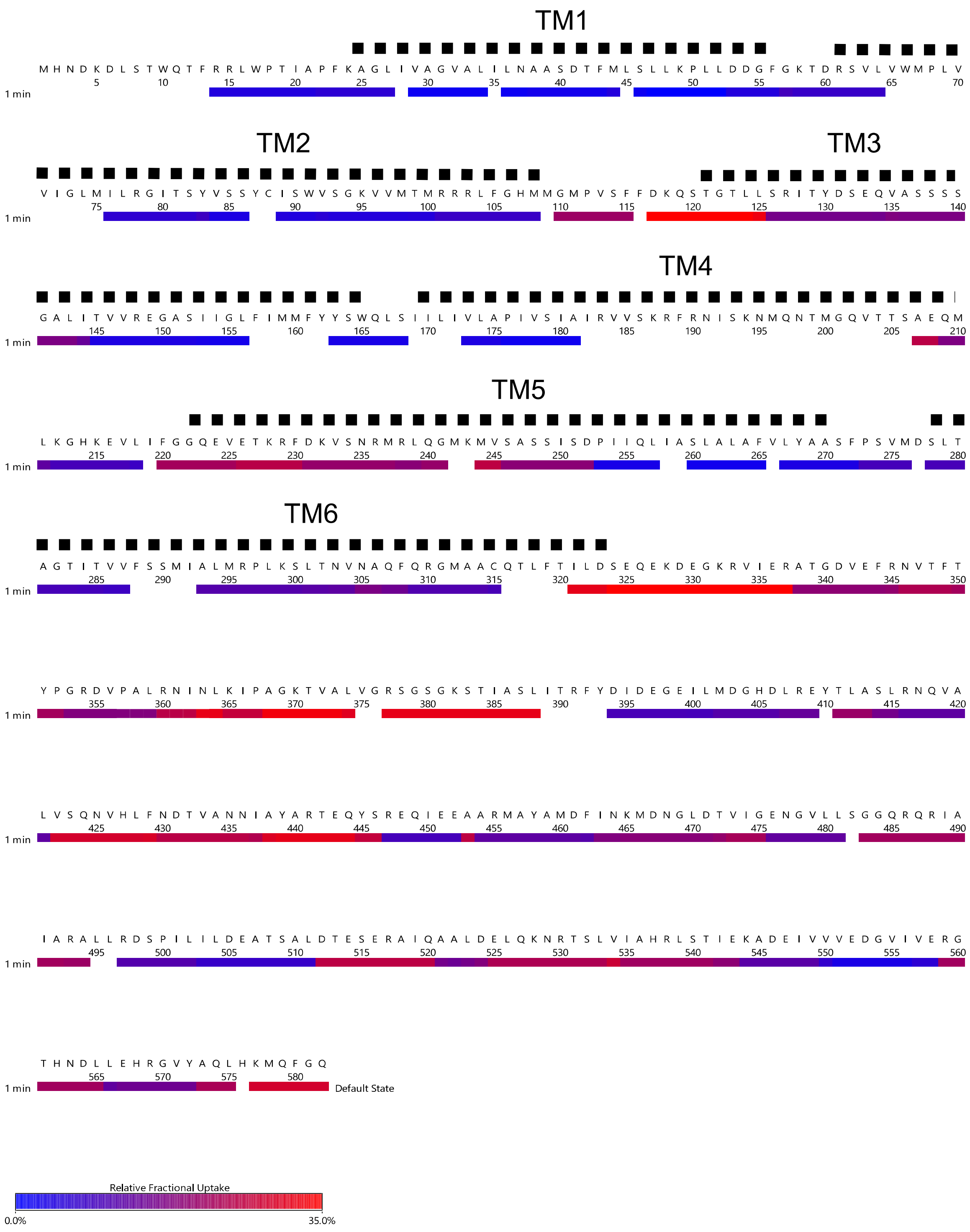

**Figure S13 – MsbA multi-pass HDX-MS heat map.** Heat maps for 1min timepoint of MsbA using cyclic multi-pass. Deuterium uptake is shown as relative fractional uptake, with the uptake of each residue being averaged across redundant peptides. Dashed black lines above the protein sequence represent known transmembrane domains/topology.

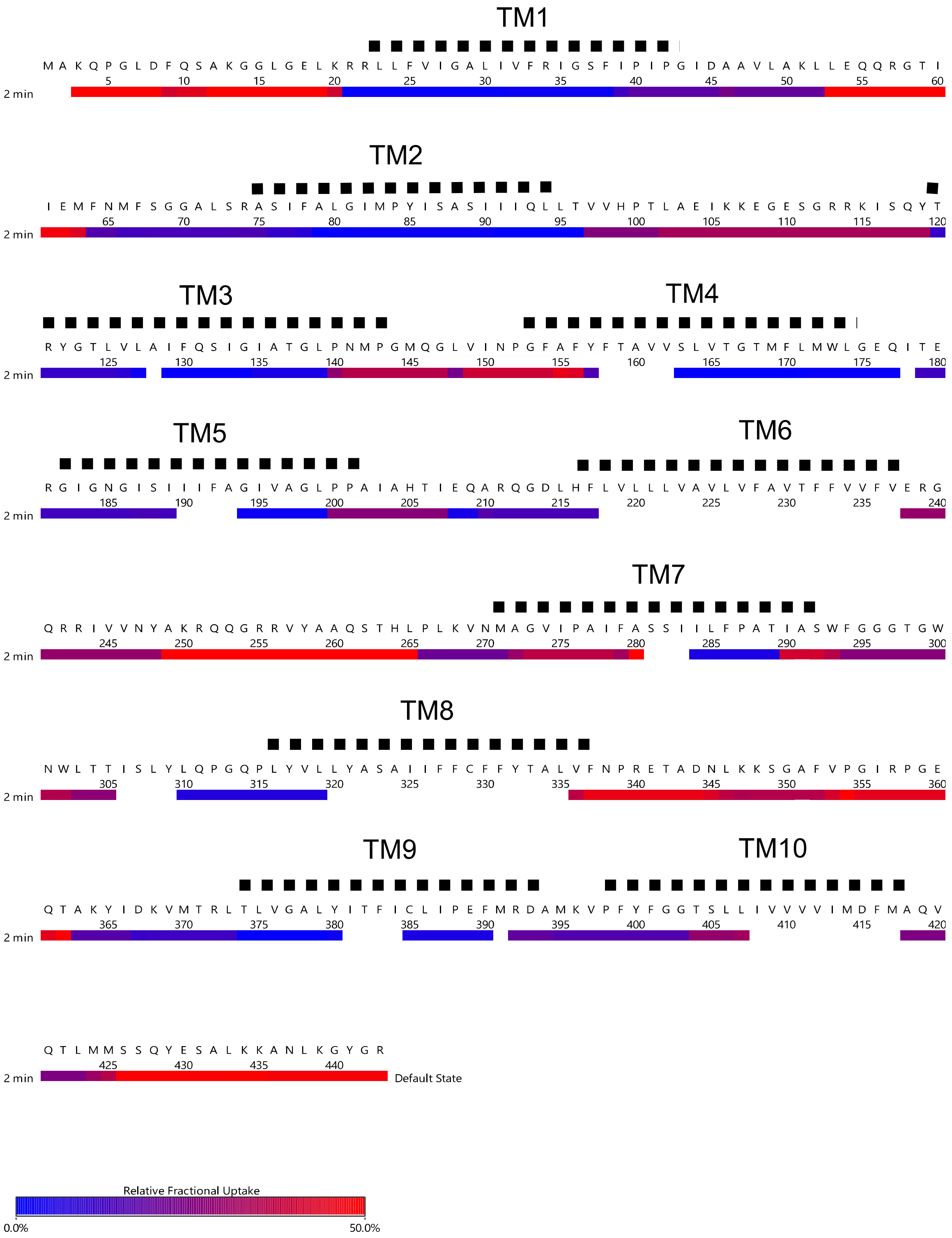


**Figure S14 – SecY 1-pass HDX-MS heat map.** Heat maps for 1min timepoint of SecY using cyclic 1-pass. Deuterium uptake is shown as relative fractional uptake, with the uptake of each residue being averaged across redundant peptides. Dashed black lines above the protein sequence represent known transmembrane domains/topology.

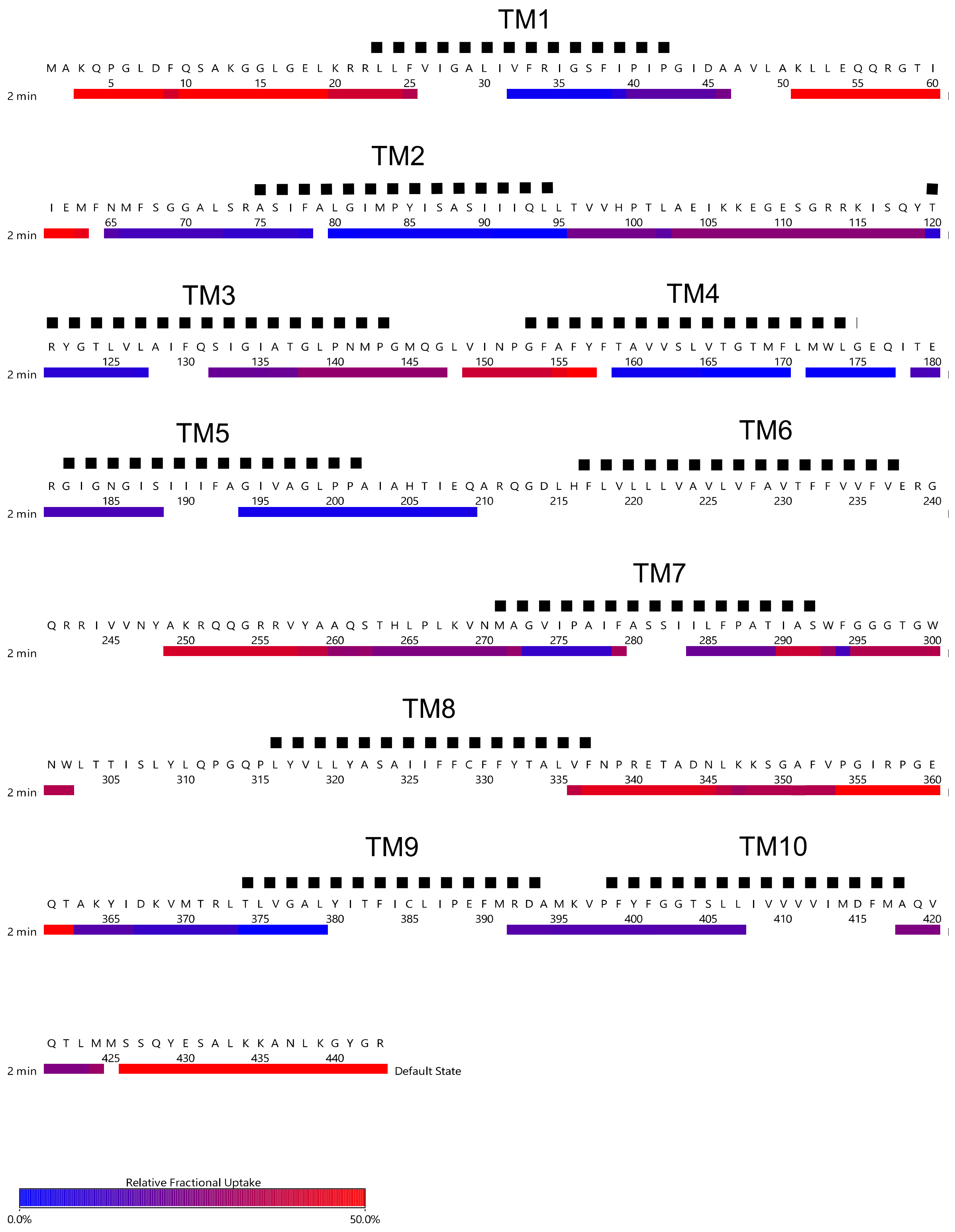

**Figure S15 – SecY multi-pass HDX-MS heat map.** Heat maps for 1min timepoint of SecY using cyclic multi-pass. Deuterium uptake is shown as relative fractional uptake, with the uptake of each residue being averaged across redundant peptides. Dashed black lines above the protein sequence represent known transmembrane domains/topology.

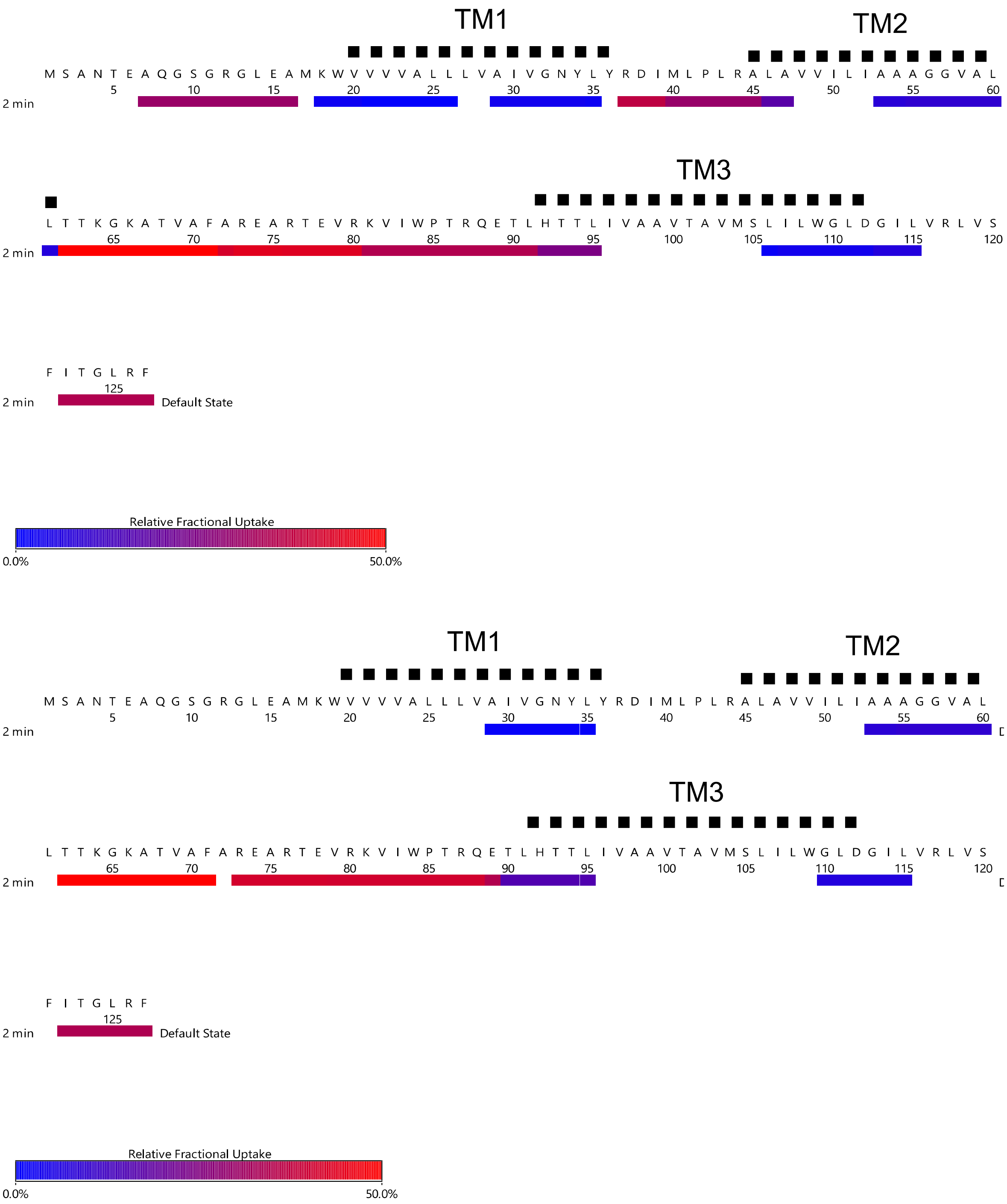


**Figure S16 – SecE 1-pass and multi-pass HDX-MS heat maps.** Heat maps for 1min timepoint of SecE using 1-pass (top) and multi-pass (bottom). Deuterium uptake is shown as relative fractional uptake, with the uptake of each residue being averaged across redundant peptides. Black lines above the protein sequence represent known membrane topology.

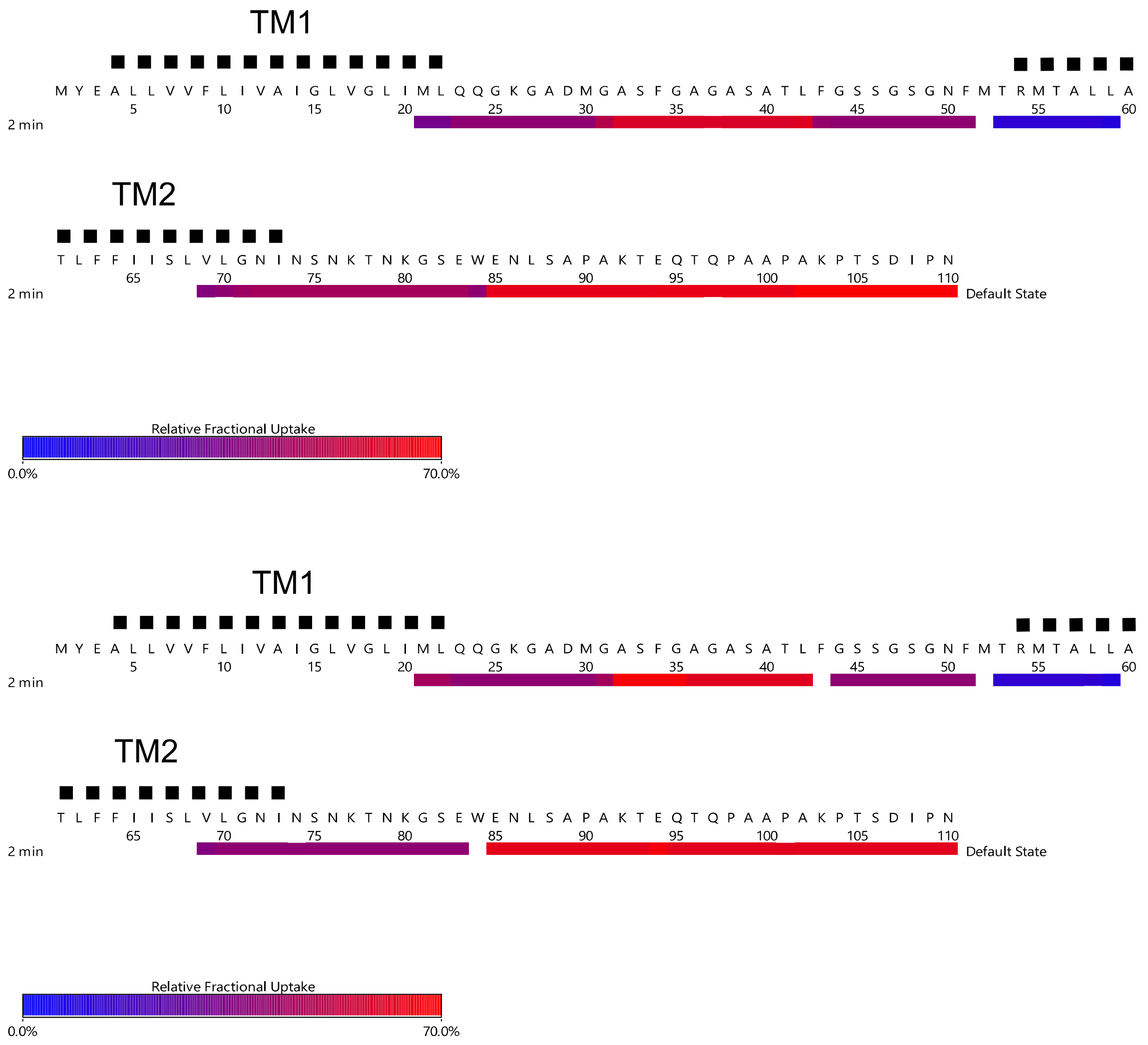


**Figure S17 – SecG 1-pass and multi-pass HDX-MS heat maps.** Heat maps for 1min timepoint of SecG using 1-pass (top) and multi-pass (bottom). Deuterium uptake is shown as relative fractional uptake, with the uptake of each residue being averaged across redundant peptides. Black lines above the protein sequence represent known membrane topology.

**Table S1 – SYNAPT G2-Si vs Cyclic IMS peptide mapping overview.** Number of peptides, sequence coverage and redundancy for SMO, XylE, MsbA SecYEG after manual curation of non-deuterated peptide mapping data using SYNAPT G2-Si, Cyclic 1-pass, and Cyclic multi-pass. Cyclic multi-pass (11-14) refers to cyclic multi-pass peptide mapping using a manual defined drift FWHM trendline with driftFWHMstart set at 11 and driftFWHMend set at 14. Cyclic 1-pass + multi-pass (11-14) refers to the combined peptide output from both peptide mapping experiments.

| | **SMO** | | | **XylE** | | | **MsbA** | | | **SecYEG** | | |
|---|---|---|---|---|---|---|---|---|---|---|---|---|
| | **Peptides** | **Coverage (%)** | **Redundanc y** | **Peptide s** | **Coverage (%)** | **Redundanc y** | **Peptides** | **Coverage (%)** | **Redundanc y** | **Peptides** | **Coverage (%)** | **Redundanc y** |
| **SYNAPT G2-Si** | 73 | 65 | 2.2 | 179 | 89 | 5.2 | 181 | 88 | 4.4 | 218 | 84 | 4.9 |
| **Cyclic 1-pass** | 178 | 86 | 3.4 | 236 | 90 | 5.7 | 267 | 92 | 5.7 | 270 | 84 | 5.7 |
| **Cyclic Multi-pass** | 125 | 69 | 3.3 | 197 | 87 | 5.2 | 168 | 89 | 3.8 | 104 | 60 | 3.0 |
| **Cyclic Multi-pass (11-14)** | 177 | 82 | 3.8 | 230 | 91 | 5.9 | 224 | 89 | 4.8 | 133 | 70 | 3.0 |
| **Cyclic 1-pass + muti-pass (11-14)** | 235 | 89 | 4.5 | 323 | 96 | 7.8 | 335 | 92 | 7.1 | 286 | 84 | 6.0 |

**Table S2 – Cyclic 1-pass vs Cyclic 1-pass/multi-pass HDX-MS overview.** Number of peptides, sequence coverage and redundancy for SMO, XylE, MsbA and SecYEG after manual curation of HDX-MS data using standalone cyclic 1-pass and cyclic 1-pass+multi-pass reference peptide databases. Peptide retention refers to the percentage of peptides retained after deuteration relative to non-deuterated peptide maps.

| | **SMO** | | | | **XylE** | | | | **MsbA** | | | | **SecYEG** | | | |
|---|---|---|---|---|---|---|---|---|---|---|---|---|---|---|---|---|
| | **Peptides** | **Retenti on (%)** | **Covera ge (%)** | **Redu ndanc y** | **Peptide s** | **Retenti on (%)** | **Covera ge (%)** | **Redu ndanc y** | **Peptide s** | **Retenti on (%)** | **Covera ge (%)** | **Redu ndanc y** | **Peptide s** | **Retenti on (%)** | **Covera ge (%)** | **Redun dancy** |
| **Cyclic 1-pass** | 154 | 87 | 85 | 3.1 | 215 | 91.1 | 89 | 5.6 | 247 | 92 | 92 | 5.2 | 264 | 97.8 | 84 | 5.5 |
| **Cyclic 1-pass + muti-pass (11-14** | 213 | 91 | 88 | 4.2 | 298 | 92.3 | 95 | 7.3 | 311 | 93 | 92 | 6.6 | 28 | 97.2 | 84 | 5.8 |

# Supporting Information References

(1) Ho, H.; Miu, A.; Alexander, M. K.; Garcia, N. K.; Oh, A.; Zilberleyb, I.; Reichelt, M.; Austin, C. D.; Tam, C.; Shriver, S.; et al. Structural basis for dual-mode inhibition of the ABC transporter MsbA. *Nature* **2018**, *557*, 196-201. DOI: 10.1038/s41586-018-0083-5.

(2) Jia, R.; Bradshaw, R. T.; Calvaresi, V.; Politis, A. Integrating Hydrogen Deuterium Exchange–Mass Spectrometry with Molecular Simulations Enables Quantification of the Conformational Populations of the Sugar Transporter XylE. *J. AM. Chem. Soc.* **2023**, *145*, 7768-7779. DOI: 10.1021/jacs.2c06148.

(3) Collinson, I.; Breyton, C.; Duong, F.; Tziatzios, C.; Schubert, D.; Or, E.; Rapoport, T.; Kühlbrandt, W. Projection structure and oligomeric properties of a bacterial core protein translocase. *EMBO J.* **2001**, *20*, 2462-2471. DOI: 10.1093/emboj/20.10.2462.
